# Molecular trade-offs in RNA ligases affected the modular emergence of complex ribozymes at the origin of life

**DOI:** 10.1098/rsos.170376

**Published:** 2017-09-20

**Authors:** Nisha Dhar, Marc S. Weinberg, Richard E. Michod, Pierre M. Durand

**Affiliations:** 1 Department of Molecular Medicine, University of the Witwatersrand, Johannesburg, South Africa; 2 Antiviral Gene Therapy Research Unit, University of the Witwatersrand, Johannesburg, South Africa; 3 Evolutionary Studies Institute, University of the Witwatersrand, Johannesburg, South Africa; 4 The Scripps Research Institute, La Jolla, CA, USA; 5 Department of Ecology and Evolutionary Biology, University of Arizona, Tucson, AZ, USA

**Keywords:** origin of life, catalytic RNA, complexity, molecular trade-off

## Abstract

In the RNA world hypothesis complex, self-replicating ribozymes were essential. For the emergence of an RNA world, less is known about the early processes that accounted for the formation of complex, long catalysts from small passively formed molecules. The functional role of small sequences has not been fully explored and, here, a possible role for smaller ligases is demonstrated. An established RNA polymerase model, the R18, was truncated from the 3′ end to generate smaller molecules. All the molecules were investigated for self-ligation functions with a set of oligonucleotide substrates without predesigned base pairing. The smallest molecule that exhibited self-ligation activity was a 40-nucleotide RNA. It also demonstrated the greatest functional flexibility as it was more general in the kinds of substrates it ligated to itself although its catalytic efficiency was the lowest. The largest ribozyme (R18) ligated substrates more selectively and with greatest efficiency. With increase in size and predicted structural stability, self-ligation efficiency improved, while functional flexibility decreased. These findings reveal that molecular size could have increased from the activity of small ligases joining oligonucleotides to their own end. In addition, there is a size-associated molecular-level trade-off that could have impacted the evolution of RNA-based life.

## 1. Introduction

We adopt the hypothesis of an RNA world at the origin of life [1,2]. RNA molecules 40–50 nucleotides in length could have formed spontaneously on montmorillonite clay by passive chemical processes without the need for biological catalysts [3,4]. However, to overcome the error rate of passive replication, an enzymatic replication process was essential. There is evidence to suggest that sets of RNA ligases and recombinases could have mutually self-assembled [5–7]. In addition, engineered and *in vitro* evolved RNA polymerases demonstrate the ability to copy external specific templates [8–10]. However, the ligases, recombinases and polymerases needed for catalytic replication are large in size; generally much larger than the ones that emerged passively in prebiotic conditions. How these complex catalysts emerged from 40 to 50 nucleotide oligomers is not clear.

In addition, a limitation of the recombination processes that may have facilitated the assembly of complex catalysts from short precursor strands [11,12] was the requirement of partial complementarity between sequences. In the pool of early RNA molecules, randomized sequences would have been much more numerous. The limitation of the required complementary substrates in the molecular neighbourhood of the catalysts could have been a constraint for the self-assembly reactions. Such catalytic reactions may have been facilitated by an RNA polymerase that would have generated the constituent partially complementary strands as a theoretical model suggested [13]. The existence of an RNA polymerase, therefore, would have been imperative for not only its own replication that may have used chemistry similar to ligases previously identified [2] but also for the feasibility of the self-assembly processes. The evolved RNA polymerases are, however, much larger in size themselves. This raises the question: what processes could have accounted for increases in molecular size at the stage of small molecules (not specifically related) for larger molecules like polymerases to emerge? We investigated this process using R18, an established exemplar RNA polymerase [8]. The R18 polymerase (R18) at the 5′ end is composed of a minor mutational variant of the Class I ligase core active region and an accessory domain at the 3′ end essential for polymerization efficiency [8]. Truncation of R18 from the 3′ end could impact polymerization function and efficiency, but it remains to be identified to what extent the truncations could impact the ligation function. The objective was to examine R18 and its truncated molecules for self-ligation activity and determine how shorter RNA ligases may have played a role. Furthermore, the ligases were examined for relationships between their size, ligation flexibility and efficiency.

## 2. Results and discussion

### 2.1. Model system

**Figure 1.**
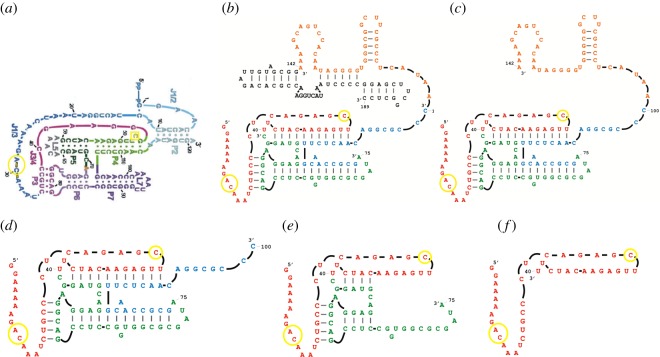
Class I ligase, R18 polymerase (R18) and truncated molecules of R18. (*a*) Secondary structure of a minor variant of parental Class I ligase (taken from [14]). The nucleotide C (marked with a yellow box) and nucleotides A and C (marked with yellow circles) formed the active site for ligation activity. (*b*) Secondary structure of R18 (redrawn based on [8]). At the 5′end is the active ligase core (in red, green and blue) and at the 3′end is the accessory domain (in orange and black). Colours depict the truncated segments from the 3′end of R18. (*c–f*) The truncated molecules of R18 used in this study. (*c*, *d*, *e* and *f*) represent structures of R18-T1, R18-T2, R18-T3 and R18-T4, respectively, for illustration only and do not depict their secondary structures. Three critical residues essential for ligation activity are marked in yellow in all the structures.

R18 polymerase (composed of a ligase core) was truncated from the 3′ end (because the 5′ region is more crucial for ligation activity) by a stepwise deletion of the structural segments (based on R18 known secondary structure). All the RNA molecules (R18, R18-T1, R18-T2, R18-T3 and R18-T4) used in the experiments (figure 1*b–f*) contained the 5′ region of the active ligase core with the three most essential nucleotides previously identified [14]; R18-T4 (40 nucleotides in size) was the smallest 5′ region. All the molecules were investigated for their ability to increase in size by ligating 35-nucleotide-long chimaeric oligonucleotide substrates (sequences in electronic supplementary material, table S1) to their own end (self-ligation). The study was initiated with substrate 1 (electronic supplementary material, table S1) that was previously used for the *in vitro* evolution of more efficient ligases from the Class I ligase core [15]. The ligase core of R18 (sequence region in red, green and blue; figure 1*b*), however, lacks the 5′ end substrate pairing segment that was used to increase ligase reaction favourability in the previous studies. The present study, therefore, examined R18 and its truncated derivatives’ self-ligation function under no experimentally designed pairing with the rationale that the prebiotic stage of the RNA world was likely to contain heterogeneous RNA strands that might not have specific base pairing with the catalysts. Catalytic reactions that are functionally favoured with substrate complementarity is a feature of previous experimental designs that could, therefore, have been constrained. The study specifically focused on processes that accounted for increases in molecular size at that stage of the RNA world. The ligation abilities of the molecules were studied with variations in the substrate 1 sequence qualitatively (either ligations occurred or not) in the presence of excess substrate concentration, and then a time course of their activity was assessed in the presence of a limited substrate concentration.

### 2.2. Self-ligation activity

**Figure 2.**
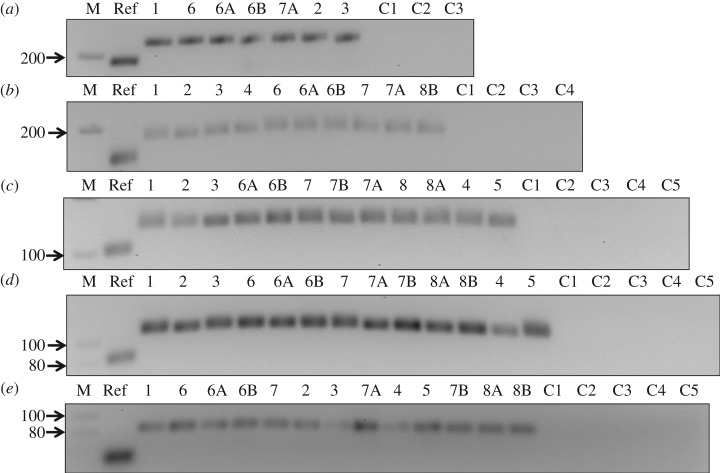
Self-ligation activity of R18 and its truncations. The PCR products from the positive ligation reactions of (*a*) R18, (*b*) R18-T1, (*c*) R18-T2, (*d*) R18-T3, and (*e*) R18-T4 RNA with the substrates (labelled on top) are shown in a 2.5% agarose gel. Lane M shows the DNA marker. Lane Ref shows the reference DNA-PCR amplified from template DNA of R18, R18-T1, R18-T2, R18-T3 and R18-T4 using forward primer F2 and reverse primers R, R1, R2, R3 and R4, respectively (details in the electronic supplementary material, figure S1). Lanes C1–C5 show the ribozyme control reactions that were set without the substrates, reverse transcribed and PCR amplified with the primer sets that were used to amplify the positive ligation reactions (primers given in the electronic supplementary material, table S2).

R18 and its truncated molecules were all able to ligate substrates to their own 5′ end (figure 2; electronic supplementary material, sections SA–SE). The smallest molecule demonstrating ligase activity was R18-T4. Based on previous structural studies of the Class I ligase variant, nucleotide C47 and the backbone phosphates of A29 and C30 are the key part of the ligase active site [14] (marked yellow in figure 1*a*). C47 was predicted to have a more direct role in the catalysis. All the molecules that were examined in this study consisted of the three essential bases (marked yellow in figure 1*b–f*) and presumably played a critical role in ligation activity. R18-T4 formed a minimal functional motif for this activity (a hairpin structure as predicted by RNAfold), although the precise mechanism of catalysis requires further study. R18-T4 represents an exemplar of the small molecules that could have existed at the very beginning of the RNA world, able to increase its own size and complexity by joining variable oligonucleotides to its own end. Naturally occurring hairpin ribozymes are quite adept at forming active catalytic sites [16,17] and this is likely to be also occurring in R18-T4. In addition, whereas previous studies showed self-ligation ability in small RNA ligases [18–20], in this study it occurred without any specifically designed pairing with the substrates. There might, however, be unintentional base pairing that could have influenced the overall ribozyme–substrate structural complex and interaction of catalytic residues that may have also resulted in the observed catalytic activity in the truncated ribozymes. Furthermore, whereas previous studies [18,19] have examined the impact of temperature and catalytic residue variation and/or truncation on the ribozyme ligation rate, this study examined the ability of truncated forms of structurally complex ligases to ligate different kinds of substrates.

### 2.3. Functional flexibility of the ribozymes

The ribozymes exhibited differential functional flexibility, which refers here to their ability to ligate different kinds of substrates to their own end. The smallest ribozyme, R18-T4 self-ligated 13 out of 24 different substrates and was most flexible in its function (table 1). With an increase in the size of the ribozyme, however, there was a gradual restriction in the kind of substrates that were preferentially self-ligated. The largest ribozyme, R18, self-ligated only 7 out of 24 different substrates and was most selective in its function. Three types of noteworthy patterns were observed. Pattern 1 (dotted leftmost region in table 1) shows a subset of substrates self-ligated by all the ribozymes in the study. This suggests that self-ligation was a preserved function in R18 as well as in its truncated molecules with limited kinds of substrates. It could have been one of the earliest functions that occurred in small oligomers before complex molecules like polymerases emerged. Pattern 2 (middle shaded region in table 1) shows a subset of substrates that were self-ligated by the shorter ribozymes (R18-T4 and R18-T3). However, with increase in the size of the ribozyme the ligation reactions within the subset progressively failed, suggesting a relationship between increase in molecular size and specificity for substrates. The flexibility of ligation in the shorter ribozymes was probably because of their less folded nature, which permitted interaction with different kinds of oligonucleotides. With increased molecular size in larger ribozymes, the degree of folding and self-pairing increased (as determined by the Gibbs free energy: R18-T4 (−3 kcal mol^−1^), R18-T3 (−18 kcal mol^−1^), R18-T2 (−31.2 kcal mol^−1^), R18-T1 (−46.7 kcal mol^−1^), R18 (−67.3 kcal mol^−1^)). The increased folding and secondary structures possibly limited their interaction with different kinds of oligonucleotides and is presumably the reason for greater specificity. This finding suggests that, in the early stages of the RNA world, the structural complexity of catalysts could have critically influenced the flexibility of the self-ligation reaction. In pattern 3 (rightmost region in table 1), a subset of substrates was not self-ligated by any of the ribozymes irrespective of their size or structural complexity. This indicates that even though the smaller ribozymes are more flexible in substrate selection, this is not completely unconstrained by substrate sequence.

**Table 1.** Self-ligation activities. (The rows represent R18 and its truncated molecules (R18-T1, R18-T2, R18-T3 and R18-T4) that were examined for self-ligation activity. The columns represent the 24 substrates that were used in the assays. The assay results are represented as a ‘+’ or ‘_’ sign. The ‘+’ sign denotes the presence of ligation (less than 35 CP value in qRT-PCR) between the ribozyme (in the row) with the substrate (in the column), and the ‘_’ sign denotes the absence of ligation (greater than 35 CP value in qRT-PCR). The regions shaded with dots, diagonal lines and no shading highlight the three different patterns observed.)

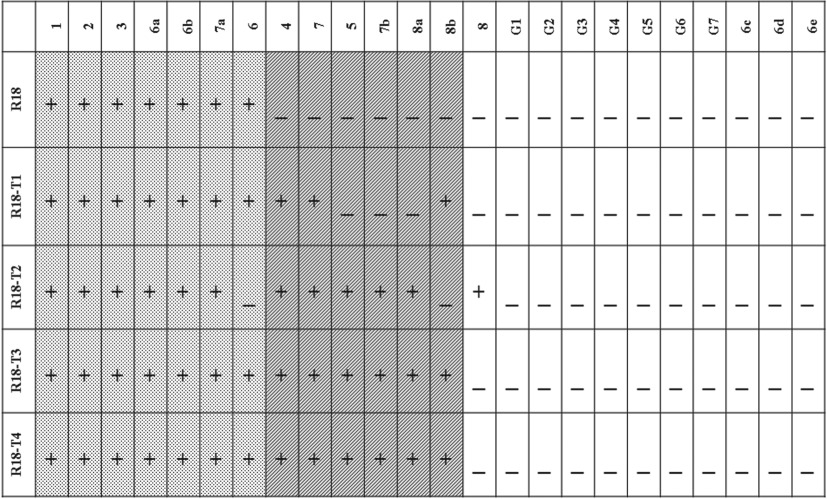

These findings provide insights into the relationship between the size and functional flexibility of the ribozymes. It also suggests that the ligation reactions occurred based on interactions beyond mere complementary template binding. In addition, the substrates were analysed for any nucleotide patterns that may have facilitated interactions with the ribozyme, enhancing the ligation reactions.

### 2.4. Substrate sequence pattern analyses

Nucleotide variability was present in all substrates used in the ligation assays (table 2, top panel). For each ribozyme, the sequence pattern of the substrates that were ligated was compared to those that were not ligated. The compared sequence patterns for R18-T4 and R18-T3 were markedly different at nucleotide positions 20, 21, 22 and 23, with a probability of greater than or equal to 60%. For R18-T2, nucleotide positions 19, 20, 21, 22 and 23 were different with a probability of greater than or equal to 50% (table 2). Substrates that were ligated included the motif AATA, while those that were not ligated included GGCG, suggesting that these differences could have played a role in substrate selection by ribozymes. This may be because of either a higher binding affinity of the ribozymes for the AATA sequence pattern over GGCG or that these specific patterns may have modified the degree of self-base pairing in substrates (electronic supplementary material, table S3), which would have affected ribozyme accessibility. For ribozymes R18-T1 and R18, there were no statistically significant differences and similarities between the compared substrate sequence patterns. It should be noted; however, that the multiple em for motif elicitation (MEME) tool is incapable of identifying gapped motifs and cannot identify structural motifs. It is possible, therefore, that R18-T1 and R18 ribozyme selection of substrates was based more on tertiary interactions.

**Table 2.** Analysis of the substrate sequence patterns for self-ligation activity of the ribozymes. (Top panel represents the sequence pattern of all the substrates used in the study. First column represents the ribozyme, the second column represents the sequence pattern of the substrates ligated by the ribozyme, and the third column represents the sequence pattern of the substrates not ligated by the ribozyme. Each logo depicts the probability (*y*-axis) of the nucleotides present at each position on the substrates (*x*-axis). The boxes outline the nucleotides that differed in the compared sequence patterns at a probability of greater than or equal to 60% (for R18-T4 and R18-T3); greater than or equal to 50% (for R18-T2).)

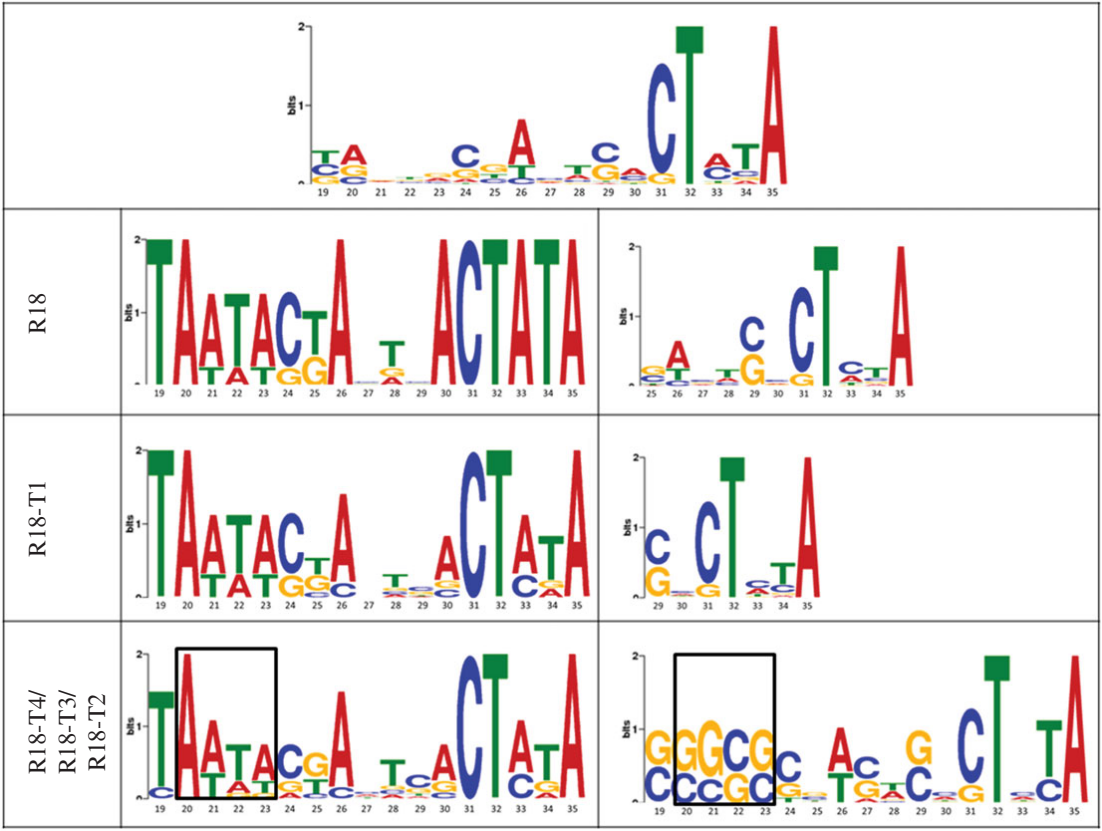

### 2.5. Self-ligation rate

Ligation reactions were analysed for efficiency (or rate of activity) and were quantified as the amount of ligated product formed over time using a customized RT-qPCR assay (see Material and methods). The reproducibility of qPCR was demonstrated by the standard curves generated for DNA copies of each ribozyme ligated to substrate 1, which showed similar CP values for equivalent copy numbers (electronic supplementary material, section SF). The quantification of all the ribozyme–substrate reactions showed an increase in product with increase in incubation time (electronic supplementary material, figures S4.3–S4.7). The efficiencies of the ribozymes with different substrates were in a narrow range, in particular the R18, R18-T1 and R18-T4 ribozymes, indicating robustness of their activity (electronic supplementary material, figure S4.8). Furthermore, the catalytic efficiencies of the ribozymes were compared based on a common set of five substrates (electronic supplementary material, figure S4.9). With each of the substrates, ribozyme efficiencies followed similar trends which showed an overall increase in the rate of ligation with increase in the size of the ribozymes. The smallest ribozyme, R18-T4, had the lowest self-ligation efficiency, and the largest, R18, had the highest efficiency. In the case of R18-T4, the low turnover of product formation could be owing to its small size, which resulted in weak binding with the substrates and susceptibility to dissociation before reaction completion. In addition, the 3′ end catalytic residues truncated from this construct could also have impacted the efficiency. An increase in the size improved the product turnover possibly because of a stronger binding of the catalysts with the substrates conferred by tertiary interactions, resulting in increased stability of the product–substrate complex for reaction completion. An exception, however, was ribozyme R18-T1, which, although larger in size than R18-T3 and R18-T2, showed a comparatively lower self-ligation rate. Based on the R18 known secondary structure, this may be because of a partially formed auxiliary domain that made unfavourable contacts with the ligase domain, resulting in a lower efficiency. In the case of R18 polymerase, the increased size formed the complete auxiliary and ligase domains which fold independently and is possibly the reason for the relatively high ligation efficiency. The comparative analysis points towards a correlation between ribozyme size and efficiency. Enhanced substrate binding and the presence of the 3′ end catalytic residues were essential for increased efficiency of the larger ribozymes. In the smaller molecules, weak substrate binding and the absence of some of the 3′ end residues compromised efficiency; however, the molecules remained minimally active for ligation by virtue of the previously identified three most essential catalytic residues [14]. The constraints that probably affected the kinetics of all the reactions in this study were the lack of designed substrate binding sites and the absence of several 2′ hydroxyl groups in the chimaeric substrates. These hydroxyl groups and substrate-binding domains promoted molecular interactions and recognition in the previous studies [18,21]. The ribozymes, however, recognized specific nucleotide patterns in the substrate sequences, which could have facilitated the reactions (discussed in the previous section). The reactions may also have been promoted by interactions with the 2′ hydroxyl groups in the four 3′ end ribonucleotides, which were closer to the ligation site. Catalysis geometry and the ribozyme–substrate interactions can be analysed in detail using structure-probing techniques, but these RNA-based techniques could not be applied to this study as the substrates were largely composed of DNA, which is a limitation here.

### 2.6. Molecular trade-offs

**Figure 3.**
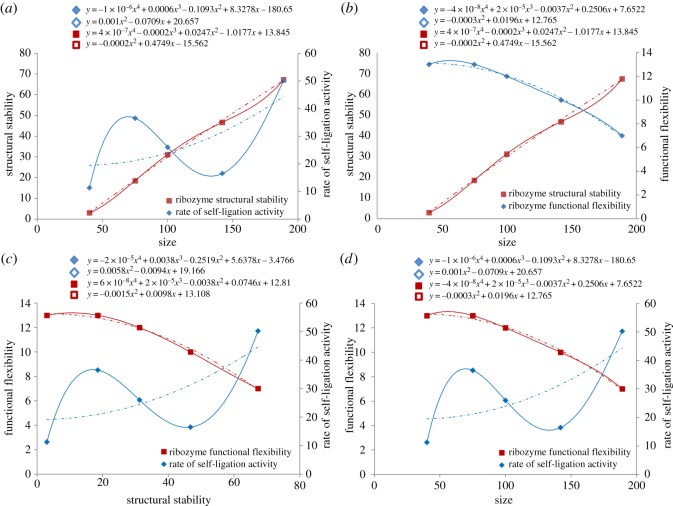
Correlation between size, predicted structural stability, functional flexibility and rate of self-ligation activity of ribozymes. Relationships were plotted for (*a*) size, structural stability and catalytic efficiency; (*b*) size, structural stability and functional flexibility; (*c*) structural stability, functional flexibility and catalytic efficiency; and (*d*) size, functional flexibility and catalytic efficiency. The size is given as the number of nucleotides in the ribozyme. Structural stability is indicated by the ribozyme's predicted Gibbs free energy (**Δ**G) using RNAfold (values of **Δ**G are negative). The functional flexibility was determined by the number of different oligonucleotide substrates that the ribozyme was able to ligate to its own end. The rate of self-ligation activity is given as the number of copies of ligated product cDNA formed per minute and is the average rate at which a ribozyme self-ligated five substrates (1, 6A, 6B, 7A, 6). Solid lines depict potential correlations and dotted lines represent the general trend. The polynomial regression model equations for each of the lines are displayed at the top of each graph; solid and empty diamonds and squares represent solid and dotted curves, respectively.

The study revealed some correlations between molecular traits such as ribozyme size, functional flexibility, catalytic efficiency and predicted structural stability (figure 3). Ribozyme size and stability did not show a significant linear relationship with efficiency; however, three ribozymes (R18-T4, R18-T2 and R18) followed a linear trend and showed a weak correlation (*R*^2^ = 0.42) (figure 3*a*). Notably, size and structural stability correlated negatively with functional flexibility (*R*^2^ = 1) (figure 3*b*), which suggests that although the smaller ribozymes had lower efficiency, they were more tolerant in self-ligating different kinds of substrates. The larger ribozymes were more efficient but more selective for the substrates. These relationships indicate that there are molecular trade-offs at play (figure 3*c,d*). Life-history trade-offs play an essential role in evolution [22–25], and this study points out that the molecular trade-offs could have also impacted the origin of RNA-based life.

## 3. Conclusion: implications for RNA evolution at the origin of life

Ligases (and related polymerases) have primarily been explored with the aim of evolving a self-replicating enzyme (2). However, while these self-replicating ribozymes are key components of a replicating RNA world, an explanation is needed for the emergence of such molecules that are much larger in size than those that formed spontaneously in the prebiotic world. This study reveals how the activity of small ligases could have led to larger, more complex molecules. The ligases exhibited differential functional flexibility and efficiency which correlated with their size and stability. The results indicate that, in the early stages of the RNA world, molecular size could have increased in a modular, stepwise fashion via the reactions of small ligases with a range of oligomers, albeit with a relatively poor efficiency. It supports the computational and theoretical predictions that assembly of larger functional molecules resulted from short RNA ligases [26,27]. The derived larger and more complex ligases developed specificity and efficiency for the kinds of substrates ligated. This trade-off could have contributed to building molecular complexity and the generation of a pool of functionally specialized molecules, which were necessary for the emergence of a self-sustained replicating system.

## 4. Material and methods

### 4.1. R18 ribozyme and truncations

The template DNA sequence for the R18 ribozyme [8] was synthesized (electronic supplementary material, figure S1-A), cloned into a pTZ57R/T plasmid vector and sequenced (Inqaba Biotechnology, Hatfield, South Africa). The cloned plasmid construct was used for amplification of the templates for R18 and the 3′ truncated molecules using the primers indicated. Amplicons were purified to homogeneity and then used for *in vitro* transcription of RNA molecules: R18, R18-T1, R18-T2, R18-T3, R18-T4 (electronic supplementary material, figure S1-B). Transcribed RNAs were purified to homogeneity in an 8%-8 M urea polyacrylamide gel. The concentration of RNAs was measured by the absorbance at 260 nm.

### 4.2. Design of the oligonucleotide substrates

Oligonucleotide substrates (35 nt in length) were synthesized as DNA–RNA chimaeras with four ribonucleotides at the 3′ end (Integrated DNA Technologies, Coralville, IA, USA) (electronic supplementary material, table S1). Substrates varied at the 3′ end (positions 19–34) except for the last ribonucleotide at the 3′ end, which was identical in all substrates. The 5′ end substrate segment (positions 1–18) was also kept constant in all substrates (with the exception of substrates 2, 3, 4 and 5) because this region was used as the generic primer-binding region for the detection of ribozyme catalytic activity (see below). There was no specifically designed complementarity of the substrates with the ribozymes. The synthesized substrates were dissolved in nuclease-free water (Sigma-Aldrich, St. Louis, MO, USA) to a stock concentration of 100 µM.

### 4.3. Detection of self-ligation activity of R18 and its truncated molecules

All the gel-purified ribozymes were PCR amplified with primers F1 and R for R18, F1 and R1 for R18-T1, F1 and R2 for R18-T2, F1 and R3 for R18-T3, and F1 and R4 for R18-T4 (electronic supplementary material, section S1) to verify the absence of non-specific amplification prior to using them in the ligation assay. The purified RNAs were assayed for self-ligation activity with each of the oligonucleotide substrates in excess concentration. The reaction buffer was composed of 25 mM MgCl_2,_ 50 mM KCl, 4 mM DTT, 50 mM EPPS (pH 8.2) in nuclease-free water. RNA (2 µM final concentration) was incubated in nuclease-free water at 80°C for 1 min, and then cooled to 37°C for 5 min. This was followed by simultaneous addition of reaction buffer and oligonucleotide substrate (5 µM final concentration), and the reaction was incubated in a total volume of 20 µl at 37°C for 40 min. At the end of the incubation period, 25 pmol of the primer complementary to the 3′ end of the RNA (electronic supplementary material, table S2), 0.4 mM of each dNTP and 200 units Superscript III reverse transcriptase (Invitrogen, Carlsbad, CA, USA) were added to the above reaction mixture and incubated in a total volume of 25 µl at 55°C for 30 min. After incubation, 5 µl was removed from the reaction and amplified by PCR using primers complementary to the 5′ end of the oligonucleotide substrate and the 3′ end of the RNA (electronic supplementary material, table S2). Electronic supplementary material, sections S2 and S3 provide schematic representation of the assay and details of the PCR conditions. The ligation reaction was detected by the size of the amplicon on a 2.5% agarose gel. Negative controls were set up by incubating the RNA without the addition of oligonucleotide substrate in the reaction buffer at 37°C for 40 min, and then they were reverse transcribed at 55°C for 30 min. The control reactions were PCR amplified with the primers that were used for detection of self-ligation activity of ribozymes (electronic supplementary material, table S2). The ligated product was purified to homogeneity and cloned into pTZ57R/T vector using the InsTAclone PCR cloning kit. Plasmid DNA from a single bacterial colony of the clones was purified and sequenced (Inqaba Biotechnology). The self-ligation reaction was confirmed by sequence alignment using the EMBOSS-Needleman–Wunsch algorithm with default parameters.

### 4.4. Substrate sequence analyses

Comparative analysis of the substrates was performed using MEME–Multiple Em for Motif Elicitation Suite 4.10.0 [28]. Based on the default nucleotide probability matrices in MEME, potential sequence patterns in the substrates that could be associated with positive or negative ligation reactions by the ribozymes were identified. First, the varied 3′ regions of all the substrates were aligned to confirm nucleotide variability at each position. Substrates 2, 3, 4 and 5 were excluded from the analysis because their 5′ end also varied (unlike all the other substrates). For each ribozyme, substrate sequences were grouped into two categories: substrates that were ligated (positive) and those not ligated (negative). The varied 3′ regions of the positive group were aligned in the 5′ to 3′ direction and analysed for the representative sequence pattern. Similarly, the substrates in the negative group were examined. The sequence patterns from the two groups were compared for nucleotides that occurred at a probability of greater than or equal to 50%.

### 4.5. Prediction of ribozyme stability and substrate structures

The structures of the substrates were predicted using mfold RNA folding form [29]. The structural stability and degree of secondary structure of the ribozymes were predicted with RNAfold; quantified using the predicted minimum free energy structure and thermodynamic stability (Gibbs free energy) [30].

### 4.6. Quantitative reverse transcription polymerase chain reaction analysis of ribozyme self-ligation activity

The standard technique for quantification of ribozyme assays entails incubation of P^32^ radiolabelled RNA or substrate and separation of products on a polyacrylamide gel. The rate of reaction using phosphorimaging is determined as the fraction of the radio-labelled reactant converted into a product over a period of time. However, under the given experimental conditions, the products were not detected using phosphorimaging, possibly owing to the low ribozyme efficiency. Quantification was, therefore, performed using the reverse transcription-quantitative real-time PCR (RT-qPCR), which is highly specific and a more sensitive quantitative detection of RNA. Specificity is conferred at three levels: via two PCR primers and a probe. A TaqMan probe was synthesized (Life technologies, Carlsbad, USA, USA) specific to a region common in the cDNA sequences of all the ribozymes. The sequence of the probe was 5′-GGAAAAAGACAAATCTGCCC-3′. The probe sequence included a fluorescent dye 6-carboxyfluorescein (FAM) at the 5′ end. The 3′ end consisted of a non-fluorescent quencher conjugated to a major groove binder moiety. The sequences of the primers are given in the electronic supplementary material, table S2. Real-time quantitative PCR analysis of known DNA copies of each ribozyme ligated to substrate 1 was performed to generate the standard curves. The method offered detection sensitivity of femtograms (fg) of the transcripts and amplification sensitivity down to 10 copies.

A time course analysis for each of the ribozymes that reacted with substrates 1, 6, 7, 8, 6a, 6b, 6c, 7a, 7b, 8a and 8b was performed. The rates of the ribozyme activity were not studied with substrates 2, 3, 4 and 5 because the primer sequences for detection of the ligation product were different from the ones used for generation of the standard curves. The reactions were set up as described earlier, except that the final concentration of the purified ribozyme was 1 µM with limiting substrate concentration (100 nM). Incubation was performed at 37°C with eight time points ranging from 5 to 40 min, set up in different reaction tubes. The reactions were stopped by snap-freezing in liquid nitrogen. After completion of all the time points, tubes were transferred on ice and simultaneously reverse transcribed for the maximum duration of time as described earlier. Two negative controls were included; one of the controls was set up by incubating the ribozyme without the addition of oligonucleotide substrate in the reaction buffer at 37°C for 40 min, with reverse transcription at 55°C for 30 min. The second control was set up by incubating the ribozyme with the addition of oligonucleotide substrate in the reaction buffer at 37°C for 40 min, but without reverse transcription. After incubation, 1 µl from each reaction was added to a 19 µl PCR set-up consisting of 5 pmoles of the designed probe along with 12.5 pmoles each of forward primer (specific to the 5′ end of the substrate), and reverse primer was specific to a common region in all the ribozymes’ template DNA sequences, i.e. the 3′ end of R18-T4 ribozyme template (to eliminate any PCR bias owing to primer binding regions). The reactions were amplified using 10 µl of TaqMan gene expression master mix (Applied Biosystems, Foster City, CA, USA), and a real-time quantitative PCR analysis was performed on a Roche Lightcycler v. 2 (Roche Diagnostics GmbH, Penzberg, Germany). Electronic supplementary material, section S4.1 provides PCR conditions.

### 4.7. Determination of the rate of ribozyme self-ligation activity

The CP value of the reaction at each of the eight time points was obtained and the number of cDNA copies was quantified from the standard curves (electronic supplementary material, section S4.2). The reactions in which the PCR product was below the CP threshold limit of the assay were considered failed ligations (CP value of greater than 35 was the threshold for the absence of DNA as quantified from the standard curves in the electronic supplementary material, section SF). A graph of cDNA copies quantified in the reaction was plotted against the duration of incubation. The rate of reaction was determined from the slope of the graph and is reported as the copies of ligated product cDNA formed per minute. The RT-qPCR method employed in this study was an indirect method of estimation of the reaction products after a given period of time. As this method involved two additional enzymatic steps, the ‘rate of reaction’ by standard definitions was not applied. However, for the purpose of comparison of the activities of R18 and its truncated molecules, the term ‘rate’ or ‘efficiency’ has been used as a surrogate for the amount of ligated product formed over a period of time.

### 4.8. Data analysis

The correlation between data variables was analysed using a polynomial regression model (Microsoft Excel). A fitted graph (solid line in figure 3) and a general trend line (dotted line in figure 3) were plotted.

## Supplementary Material

Supplementary Tables of sequence data, meme analyses and RNA structures

## Supplementary Material

Supplementary Figures providing detailed experimental protocols and methodologies

## Data accessibility

All data presented in this study are available in the electronic supplementary material, files S1 and S2 associated with this manuscript.

## References

[1] Gilbert W 1986 Origin of life: the RNA world. Nature 319, 618 (doi:10.1038/319618a0)

[2] Joyce GF 2007 Forty years of *in vitro* evolution. Angew. Chem. 46, 6420–6436. (doi:10.1002/anie.200701369) 17634987 10.1002/anie.200701369

[3] Ferris JP 2002 Montmorillonite catalysis of 30–50 mer oligonucleotides: laboratory demonstration of potential steps in the origin of the RNA world. Orig. Life Evol. Biosph. 32, 311–332. (doi:10.1023/A:1020543312109) 12458736 10.1023/a:1020543312109

[4] Ferris JP 2006 Montmorillonite-catalysed formation of RNA oligomers: the possible role of catalysis in the origins of life. Phil. Trans. R. Soc. B 361, 1777–1786; discussion 1786 (doi:10.1098/rstb.2006.1903) 17008218 10.1098/rstb.2006.1903 PMC1664692

[5] Lincoln TA, Joyce GF 2009 Self-sustained replication of an RNA enzyme. Science 323, 1229–1232. (doi:10.1126/science.1167856) 19131595 10.1126/science.1167856 PMC2652413

[6] Vaidya N, Manapat ML, Chen IA, Xulvi-Brunet R, Hayden EJ, Lehman N 2012 Spontaneous network formation among cooperative RNA replicators. Nature 491, 72–77. (doi:10.1038/nature11549) 23075853 10.1038/nature11549

[7] Vaidya N, Walker SI, Lehman N 2013 Recycling of informational units leads to selection of replicators in a prebiotic soup. Chem. Biol. 20, 241–252. (doi:10.1016/j.chembiol.2013.01.007) 23438753 10.1016/j.chembiol.2013.01.007

[8] Johnston WK, Unrau PJ, Lawrence MS, Glasner ME, Bartel DP 2001 RNA-catalyzed RNA polymerization: accurate and general RNA-templated primer extension. Science 292, 1319–1325. (doi:10.1126/science.1060786) 11358999 10.1126/science.1060786

[9] Wochner A, Attwater J, Coulson A, Holliger P 2011 Ribozyme-catalyzed transcription of an active ribozyme. Science 332, 209–212. (doi:10.1126/science.1200752) 21474753 10.1126/science.1200752

[10] Attwater J, Wochner A, Holliger P 2013 In-ice evolution of RNA polymerase ribozyme activity. Nat. Chem. 5, 1011–1018. (doi:10.1038/nchem.1781) 24256864 10.1038/nchem.1781 PMC3920166

[11] Hayden EJ, Lehman N 2006 Self-assembly of a group I intron from inactive oligonucleotide fragments. Chem. Biol. 13, 909–918. (doi:10.1016/j.chembiol.2006.06.014) 16931340 10.1016/j.chembiol.2006.06.014

[12] Hayden EJ, von Kiedrowski G, Lehman N 2008 Systems chemistry on ribozyme self-construction: evidence for anabolic autocatalysis in a recombination network. Angew. Chem. 47, 8424–8428. (doi:10.1002/anie.200802177) 18780409 10.1002/anie.200802177

[13] Meyer AJ, Ellefson JW, Ellington AD 2012 Abiotic self-replication. Acc. Chem. Res. 45, 2097–2105. (doi:10.1021/ar200325v) 22891822 10.1021/ar200325v

[14] Shechner DM, Grant RA, Bagby SC, Koldobskaya Y, Piccirilli JA, Bartel DP 2009 Crystal structure of the catalytic core of an RNA-polymerase ribozyme. Science 326, 1271–1275. (doi:10.1126/science.1174676) 19965478 10.1126/science.1174676 PMC3978776

[15] Wright MC, Joyce GF 1997 Continuous *in vitro* evolution of catalytic function. Science 276, 614–617. (doi:10.1126/science.276.5312.614) 9110984 10.1126/science.276.5312.614

[16] Puerta-Fernández E, Romero-López C, Barroso-delJesus A, Berzal-Herranz A 2003 Ribozymes: recent advances in the development of RNA tools. FEMS Microbiol. Rev. 27, 75–97. (doi:10.1016/S0168-6445(03)00020-2) 12697343 10.1016/S0168-6445(03)00020-2

[17] Svoboda P, Di Cara A 2006 Hairpin RNA: a secondary structure of primary importance. Cell. Mol. Life Sci. 63, 901–908. (doi:10.1007/s00018-005-5558-5) 16568238 10.1007/s00018-005-5558-5 PMC11136179

[18] Vlassov AV, Johnston BH, Landweber LF, Kazakov SA 2004 Ligation activity of fragmented ribozymes in frozen solution: implications for the RNA world. Nucleic Acids Res. 32, 2966–2974. (doi:10.1093/nar/gkh601) 15161960 10.1093/nar/gkh601 PMC419604

[19] Robertson MP, Hesselberth JR, Ellington AD 2001 Optimization and optimality of a short ribozyme ligase that joins non-Watson-Crick base pairings. RNA 7, 513–523. (doi:10.1017/S1355838201002199) 11345430 10.1017/s1355838201002199 PMC1370105

[20] Landweber LF, Pokrovskaya ID 1999 Emergence of a dual-catalytic RNA with metal-specific cleavage and ligase activities: the spandrels of RNA evolution. Proc. Natl Acad. Sci. USA 96, 173–178. (doi:10.1073/pnas.96.1.173) 9874791 10.1073/pnas.96.1.173 PMC15112

[21] Muller UF, Bartel DP 2003 Substrate 2′-hydroxyl groups required for ribozyme-catalyzed polymerization. Chem. Biol. 10, 799–806. (doi:10.1016/S1074-5521(03)00171-6) 14522050 10.1016/s1074-5521(03)00171-6

[22] Flatt T, Heyland A 2011 Mechanisms of life history evolution: the genetics and physiology of life history traits and trade-offs. Oxford, UK: Oxford University Press.

[23] Stearns SC 1992 The evolution of life histories. Oxford, UK: Oxford University Press.

[24] Roff DA 2002 Life history evolution. Sunderland, MA: Sinauer Associates.

[25] Roff DA 2007 Contributions of genomics to life-history theory. Nat. Rev. Genet. 8, 116–125. (doi:10.1038/nrg2040) 17230198 10.1038/nrg2040

[26] Manrubia SC, Briones C 2007 Modular evolution and increase of functional complexity in replicating RNA molecules. RNA 13, 97–107. (doi:10.1261/rna.203006) 17105993 10.1261/rna.203006 PMC1705761

[27] Briones C, Stich M, Manrubia SC 2009 The dawn of the RNA world: toward functional complexity through ligation of random RNA oligomers. RNA 15, 743–749. (doi:10.1261/rna.1488609) 19318464 10.1261/rna.1488609 PMC2673073

[28] Bailey TL, Boden M, Buske FA, Frith M, Grant CE, Clementi L, Ren J, Li WW, Noble WS 2009 MEME SUITE: tools for motif discovery and searching. Nucleic Acids Res. 37, W202–W208. (doi:10.1093/nar/gkp335) 19458158 10.1093/nar/gkp335 PMC2703892

[29] Zuker M 2003 Mfold web server for nucleic acid folding and hybridization prediction. Nucleic Acids Res. 31, 3406–3415. (doi:10.1093/nar/gkg595) 12824337 10.1093/nar/gkg595 PMC169194

[30] Zuker M, Stiegler P 1981 Optimal computer folding of large RNA sequences using thermodynamics and auxiliary information. Nucleic Acids Res. 9, 133–148. (doi:10.1093/nar/9.1.133) 6163133 10.1093/nar/9.1.133 PMC326673

