# Supplementary material for: Molecular trade-offs in RNA ligases affected the modular emergence of complex ribozymes at the origin of life
Source: R Soc Open Sci. 2017 Sep 20;4(9):170376. doi: 10.1098/rsos.170376 (PMC5627087; doi:10.1098/rsos.170376)
Supplement: Supplementary Tables of sequence data, meme analyses and RNA structures [file rsos170376supp1.docx]

Table S1: Oligonucleotide substrates

Substrate sequences are shown in 5’ to 3’ direction. All the sequences were DNA-RNA chimaeras with four ribonucleotides at the 3’ end (shown in bold). The rationale behind using chimeric substrates was to study the interaction of the ribozymes with different substrates that formed varying degree of secondary structures. The use of deoxyribonucleotides was to restrict the formation of tertiary structures within the substrate sequence. The use of substrate sequences composed all with ribonucleotides would have allowed tertiary structures and that was not suitable to examine the specific objectives of the study. The design of the substrates was based on a previous study (Wright and Joyce, 1997, Science, 276) that found inclusion of four ribonucleotides in the chimeric substrates optimal for the continuous in vitro evolution of a developed ligase core (Bartel and Szostak, 1993, Science, 261). Although our objectives were different but the polymerase in our study has the same ligase core (except the specifically designed substrate binding sequence at the 5’end) and therefore the study was initiated with the substrate design that was found by Wright et al to be atleast compatible for the ligation reaction. Once this substrate worked, it was varied at a few positions to examine the impact of variation in nucleotides and varying degrees of substrate secondary structures on the ribozyme reactions. We attempted to vary the nucleotides almost at every position (Table S3). The choice of the specific nucleotide varied at a position was random, however, the choice of nucleotides was made in such a way so that the substrates are either less folded or highly folded. This was done to examine the impact of degree of substrates secondary structure on the ligation activity. The substrate secondary structures were predicted using mfold (Table S4).

| **Substrates** | **Sequence in 5’ to 3’ direction** |
| --- | --- |
| Substrate 1 | CTC GAC GTC AGC CTG GAC TAA TAC GAC TCA C**UA UA** |
| Substrate 2 | GTC AAC TTC CGC ATG AAC GAA TAC TAC GCA C**UA AA** |
| Substrate 3 | CAC GAC GAC AAC CTG GTC TAA TAC GCC TCA C**GA UA** |
| Substrate 4 | CTG GAT GTA AGT CTT GAA TAT ATG GAA TCG C**UC GA** |
| Substrate 5 | TAA TAC TCA TAA CGA CTA CAT GGA CCT CGC C**UC AA** |
| Substrate 6 | CTC GAC GTC AGC CTG GAC TAA TAC TAA AAA C**UA UA** |
| Substrate 7 | CTC GAC GTC AGC CTG GAC TAT ATG GAA TCG C**UC GA** |
| Substrate 8 | CTC GAC GTC AGC CTG GAC CAT GGA CCT CGC C**UC AA** |
| Substrate 6a | CTC GAC GTC AGC CTG GAC TAA TAC TAT TTA C**UA UA** |
| Substrate 6b | CTC GAC GTC AGC CTG GAC TAA TAC TAG GGA C**UA UA** |
| Substrate 6c | CTC GAC GTC AGC CTG GAC GGG GGC TAG GGA C**UA UA** |
| Substrate 7a | CTC GAC GTC AGC CTG GAC TAT ATG GAC TCA C**UA UA** |
| Substrate 7b | CTC GAC GTC AGC CTG GAC TAA TAC GAA TCG C**UC GA** |
| Substrate 8a | CTC GAC GTC AGC CTG GAC CAT GGA GAC TCA C**UA UA** |
| Substrate 8b | CTC GAC GTC AGC CTG GAC TAA TAC CCT CGC C**UC AA** |
| Substrate G1 | CTC GAC GTC AGC CTG GAC CCC CGC GAC TCC C**UC CA** |
| Substrate G2 | CTC GAC GTC AGC CTG GAC CCC CGG CTG AGC C**UC CA** |
| Substrate G3 | CTC GAC GTC AGC CTG GAC GGG GCC GAC TCC C**UC UA** |
| Substrate G4 | CTC GAC GTC AGC CTG GAC GGG GCG CTG AGC C**UC UA** |
| Substrate G5 | CTC GAC GTC AGC CTG GAC CGG CGC GAC TCT C**UU UA** |
| Substrate G6 | CTC GAC GTC AGC CTG GAC CGG CGG CTG AGT G**UU UA** |
| Substrate G7 | CTC GAC GTC AGC CTG GAC GCG CCT ATA AGG G**UG CA** |
| Substrate 6d | CTC GAC GTC AGC CTG GAC GGG GGC GAC TCA C**UA UA** |
| Substrate 6e | CTC GAC GTC AGC CTG GAC CCC CCC TAC CCA C**UA UA** |

Table S2: Sequences of the primers used for detection of self-ligation activity of the ribozymes

Primers (shown in 5’ to 3’ direction) used for detection of self-ligation activity of R18 ribozyme and its truncated RNAs with the substrates (except substrates 2, 3, 4, and 5). The self-ligation activity of RNAs in case of substrates 2, 3, 4 and 5, was detected by using forward primers GTCAACTTCCGCATGAAC, CACGACGACAACCTGGTC, CTGGATGTAAGTCTTGAA, TAATACTCATAACGACTA, respectively (complementary to 5’end of the substrates, shown in 5’-3’direction) and reverse primers for respective RNAs as given in the table.

| RNA | Primer complementary to 5’ end of substrate (Forward primer) | Primer complementary to 3’ end of the RNA template (Reverse Primer) |
| --- | --- | --- |
| R18 | CTCGACGTCAGCCTGGAC | GGAGCCGAAGCTCCGGG |
| R18-T1 | CTCGACGTCAGCCTGGAC | TTTTCGTCAGGTGTTATCCCC |
| R18-T2 | CTCGACGTCAGCCTGGAC | GGGCGCCTGTTGAGAACG |
| R18-T3 | CTCGACGTCAGCCTGGAC | TATCGCGCCACCGGAGG |
| R18-T4 | CTCGACGTCAGCCTGGAC | AAGATGTTCTCAAGCTCTGAG |

Table SM: Motif Letter-Probability matrices.

The letter probability matrix represents a table of probabilities where the rows are positions in the sequence pattern and the columns are four nucleotides A, C, G, and T. Probability matrix gives the probability of each nucleotide at each position in the sequence pattern. Following are probability matrices of A. All the substrates used in MEME analysis. B. Substrates ligated (left) and not ligated by R18-T4 ribozyme (right). C. Substrates ligated (left) and not ligated by R18-T3 ribozyme (right). D. Substrates ligated (left) and not ligated by R18-T2 ribozyme (right). E. Substrates ligated (left) and not ligated by R18-T1 ribozyme (right). F. Substrates ligated (left) and not ligated by R18 ribozyme (right).

**(A)**

| **Position** | **A** | **C** | **G** | **T** |
| --- | --- | --- | --- | --- |
| **(5’ to 3’)** |  |  |  |  |
| 19 | 0 | 0.35 | 0.25 | 0.4 |
| 20 | 0.5 | 0.2 | 0.3 | 0 |
| 21 | 0.3 | 0.15 | 0.35 | 0.2 |
| 22 | 0.1 | 0.3 | 0.3 | 0.3 |
| 23 | 0.3 | 0.2 | 0.4 | 0.1 |
| 24 | 0.1 | 0.6 | 0.25 | 0.05 |
| 25 | 0.05 | 0.25 | 0.45 | 0.25 |
| 26 | 0.7 | 0.1 | 0 | 0.2 |
| 27 | 0.2 | 0.4 | 0.25 | 0.15 |
| 28 | 0.25 | 0.15 | 0.1 | 0.5 |
| 29 | 0.05 | 0.5 | 0.4 | 0.05 |
| 30 | 0.45 | 0.3 | 0.15 | 0.1 |
| 31 | 0 | 0.9 | 0.1 | 0 |
| 32 | 0 | 0 | 0 | 1 |
| 33 | 0.45 | 0.4 | 0.05 | 0.1 |
| 34 | 0.1 | 0.15 | 0.1 | 0.65 |
| 35 | 1 | 0 | 0 | 0 |

(B)

| **Position** | **A** | **C** | **G** | **T** |
| --- | --- | --- | --- | --- |
| **(5’ to 3’)** |  |  |  |  |
| 19 | 0 | 0.1111 | 0 | 0.8888 |
| 20 | 1 | 0 | 0 | 0 |
| 21 | 0.6666 | 0 | 0 | 0.3333 |
| 22 | 0.2222 | 0 | 0.1111 | 0.6666 |
| 23 | 0.6666 | 0 | 0.1111 | 0.2222 |
| 24 | 0.1111 | 0.6666 | 0.2222 | 0 |
| 25 | 0 | 0.1111 | 0.5555 | 0.3333 |
| 26 | 0.8888 | 0.1111 | 0 | 0 |
| 27 | 0.3333 | 0.3333 | 0.1111 | 0.2222 |
| 28 | 0.1111 | 0.1111 | 0.1111 | 0.6666 |
| 29 | 0.1111 | 0.5555 | 0.2222 | 0.1111 |
| 30 | 0.6666 | 0.1111 | 0.2222 | 0 |
| 31 | 0 | 1 | 0 | 0 |
| 32 | 0 | 0 | 0 | 1 |
| 33 | 0.6666 | 0.3333 | 0 | 0 |
| 34 | 0.1111 | 0 | 0.2222 | 0.6666 |
| 35 | 1 | 0 | 0 | 0 |

| **Position** | **A** | **C** | **G** | **T** |
| --- | --- | --- | --- | --- |
| **(5’ to 3’)** |  |  |  |  |
| 19 | 0 | 0.5 | 0.5 | 0 |
| 20 | 0 | 0.4 | 0.6 | 0 |
| 21 | 0 | 0.3 | 0.7 | 0 |
| 22 | 0 | 0.6 | 0.4 | 0 |
| 23 | 0 | 0.4 | 0.6 | 0 |
| 24 | 0 | 0.6 | 0.3 | 0.1 |
| 25 | 0.1 | 0.3 | 0.4 | 0.2 |
| 26 | 0.6 | 0 | 0 | 0.4 |
| 27 | 0.1 | 0.5 | 0.4 | 0 |
| 28 | 0.4 | 0.1 | 0.1 | 0.4 |
| 29 | 0 | 0.5 | 0.5 | 0 |
| 30 | 0.3 | 0.4 | 0.1 | 0.2 |
| 31 | 0 | 0.8 | 0.2 | 0 |
| 32 | 0 | 0 | 0 | 1 |
| 33 | 0.3 | 0.4 | 0.1 | 0.2 |
| 34 | 0 | 0.3 | 0 | 0.7 |
| 35 | 1 | 0 | 0 | 0 |

**(C)**

| **Position** | **A** | **C** | **G** | **T** |
| --- | --- | --- | --- | --- |
| **(5’ to 3’)** |  |  |  |  |
| 19 | 0 | 0.1111 | 0 | 0.8888 |
| 20 | 1 | 0 | 0 | 0 |
| 21 | 0.6666 | 0 | 0 | 0.3333 |
| 22 | 0.2222 | 0 | 0.1111 | 0.6666 |
| 23 | 0.6666 | 0 | 0.1111 | 0.2222 |
| 24 | 0.1111 | 0.6666 | 0.2222 | 0 |
| 25 | 0 | 0.1111 | 0.5555 | 0.3333 |
| 26 | 0.8888 | 0.1111 | 0 | 0 |
| 27 | 0.3333 | 0.3333 | 0.1111 | 0.2222 |
| 28 | 0.1111 | 0.1111 | 0.1111 | 0.6666 |
| 29 | 0.1111 | 0.5555 | 0.2222 | 0.1111 |
| 30 | 0.6666 | 0.1111 | 0.2222 | 0 |
| 31 | 0 | 1 | 0 | 0 |
| 32 | 0 | 0 | 0 | 1 |
| 33 | 0.6666 | 0.3333 | 0 | 0 |
| 34 | 0.1111 | 0 | 0.2222 | 0.6666 |
| 35 | 1 | 0 | 0 | 0 |

| **Position** | **A** | **C** | **G** | **T** |
| --- | --- | --- | --- | --- |
| **(5’ to 3’)** |  |  |  |  |
| 19 | 0 | 0.5 | 0.5 | 0 |
| 20 | 0 | 0.4 | 0.6 | 0 |
| 21 | 0 | 0.3 | 0.7 | 0 |
| 22 | 0 | 0.6 | 0.4 | 0 |
| 23 | 0 | 0.4 | 0.6 | 0 |
| 24 | 0 | 0.6 | 0.3 | 0.1 |
| 25 | 0.1 | 0.3 | 0.4 | 0.2 |
| 26 | 0.6 | 0 | 0 | 0.4 |
| 27 | 0.1 | 0.5 | 0.4 | 0 |
| 28 | 0.4 | 0.1 | 0.1 | 0.4 |
| 29 | 0 | 0.5 | 0.5 | 0 |
| 30 | 0.3 | 0.4 | 0.1 | 0.2 |
| 31 | 0 | 0.8 | 0.2 | 0 |
| 32 | 0 | 0 | 0 | 1 |
| 33 | 0.3 | 0.4 | 0.1 | 0.2 |
| 34 | 0 | 0.3 | 0 | 0.7 |
| 35 | 1 | 0 | 0 | 0 |

**(D)**

| **Position** | **A** | **C** | **G** | **T** |
| --- | --- | --- | --- | --- |
| **(5’ to 3’)** |  |  |  |  |
| 19 | 0 | 0.1428 | 0 | 0.8571 |
| 20 | 1 | 0 | 0 | 0 |
| 21 | 0.5714 | 0 | 0 | 0.4285 |
| 22 | 0.2857 | 0 | 0.1428 | 0.5714 |
| 23 | 0.5714 | 0 | 0.1428 | 0.2857 |
| 24 | 0.1428 | 0.5714 | 0.2857 | 0 |
| 25 | 0 | 0 | 0.7142 | 0.2857 |
| 26 | 1 | 0 | 0 | 0 |
| 27 | 0.2857 | 0.4285 | 0.1428 | 0.1428 |
| 28 | 0 | 0 | 0.1428 | 0.8571 |
| 29 | 0 | 0.7142 | 0.1428 | 0.1428 |
| 30 | 0.7142 | 0 | 0.2857 | 0 |
| 31 | 0 | 1 | 0 | 0 |
| 32 | 0 | 0 | 0 | 1 |
| 33 | 0.7142 | 0.2857 | 0 | 0 |
| 34 | 0 | 0 | 0.2857 | 0.7142 |
| 35 | 1 | 0 | 0 | 0 |

| **Position** | **A** | **C** | **G** | **T** |
| --- | --- | --- | --- | --- |
| **(5’ to 3’)** |  |  |  |  |
| 19 | 0 | 0.5 | 0.5 | 0 |
| 20 | 0 | 0.4 | 0.6 | 0 |
| 21 | 0 | 0.3 | 0.7 | 0 |
| 22 | 0 | 0.6 | 0.4 | 0 |
| 23 | 0 | 0.4 | 0.6 | 0 |
| 24 | 0 | 0.6 | 0.3 | 0.1 |
| 25 | 0.1 | 0.3 | 0.4 | 0.2 |
| 26 | 0.6 | 0 | 0 | 0.4 |
| 27 | 0.1 | 0.5 | 0.4 | 0 |
| 28 | 0.4 | 0.1 | 0.1 | 0.4 |
| 29 | 0 | 0.5 | 0.5 | 0 |
| 30 | 0.3 | 0.4 | 0.1 | 0.2 |
| 31 | 0 | 0.8 | 0.2 | 0 |
| 32 | 0 | 0 | 0 | 1 |
| 33 | 0.3 | 0.4 | 0.1 | 0.2 |
| 34 | 0 | 0.3 | 0 | 0.7 |
| 35 | 1 | 0 | 0 | 0 |

**(E)**

| **Position** | **A** | **C** | **G** | **T** |
| --- | --- | --- | --- | --- |
| **(5’ to 3’)** |  |  |  |  |
| 19 | 0 | 0 | 0 | 1 |
| 20 | 1 | 0 | 0 | 0 |
| 21 | 0.7142 | 0 | 0 | 0.2857 |
| 22 | 0.2857 | 0 | 0 | 0.7142 |
| 23 | 0.7142 | 0 | 0 | 0.2857 |
| 24 | 0 | 0.7142 | 0.2857 | 0 |
| 25 | 0 | 0.1428 | 0.4285 | 0.4285 |
| 26 | 0.8571 | 0.1428 | 0 | 0 |
| 27 | 0.2857 | 0.2857 | 0.1428 | 0.2857 |
| 28 | 0.1428 | 0.1428 | 0.1428 | 0.5714 |
| 29 | 0.1428 | 0.4285 | 0.2857 | 0.1428 |
| 30 | 0.7142 | 0.1428 | 0.1428 | 0 |
| 31 | 0 | 1 | 0 | 0 |
| 32 | 0 | 0 | 0 | 1 |
| 33 | 0.7142 | 0.2857 | 0 | 0 |
| 34 | 0.1428 | 0 | 0.1428 | 0.7142 |
| 35 | 1 | 0 | 0 | 0 |

| **Position** | **A** | **C** | **G** | **T** |
| --- | --- | --- | --- | --- |
| **(5’ to 3’)** |  |  |  |  |
| 19 | 0 | 0 | 0 | 0 |
| 20 | 0 | 0 | 0 | 0 |
| 21 | 0 | 0 | 0 | 0 |
| 22 | 0 | 0 | 0 | 0 |
| 23 | 0 | 0 | 0 | 0 |
| 24 | 0 | 0 | 0 | 0 |
| 25 | 0 | 0 | 0 | 0 |
| 26 | 0 | 0 | 0 | 0 |
| 27 | 0 | 0 | 0 | 0 |
| 28 | 0 | 0 | 0 | 0 |
| 29 | 0 | 0.5384 | 0.4615 | 0 |
| 30 | 0.3076 | 0.3846 | 0.1538 | 0.1538 |
| 31 | 0 | 0.8461 | 0.1538 | 0 |
| 32 | 0 | 0 | 0 | 1 |
| 33 | 0.3076 | 0.4615 | 0.0769 | 0.1538 |
| 34 | 0.0769 | 0.2307 | 0.0769 | 0.6153 |
| 35 | 1 | 0 | 0 | 0 |

**(F)**

| **Position** | **A** | **C** | **G** | **T** |
| --- | --- | --- | --- | --- |
| **(5’ to 3’)** |  |  |  |  |
| 19 | 0 | 0 | 0 | 1 |
| 20 | 1 | 0 | 0 | 0 |
| 21 | 0.8 | 0 | 0 | 0.2 |
| 22 | 0.2 | 0 | 0 | 0.8 |
| 23 | 0.8 | 0 | 0 | 0.2 |
| 24 | 0 | 0.8 | 0.2 | 0 |
| 25 | 0 | 0 | 0.4 | 0.6 |
| 26 | 1 | 0 | 0 | 0 |
| 27 | 0.2 | 0.4 | 0.2 | 0.2 |
| 28 | 0.2 | 0 | 0.2 | 0.6 |
| 29 | 0.2 | 0.4 | 0.2 | 0.2 |
| 30 | 1 | 0 | 0 | 0 |
| 31 | 0 | 1 | 0 | 0 |
| 32 | 0 | 0 | 0 | 1 |
| 33 | 1 | 0 | 0 | 0 |
| 34 | 0 | 0 | 0 | 1 |
| 35 | 1 | 0 | 0 | 0 |

| **Position** | **A** | **C** | **G** | **T** |
| --- | --- | --- | --- | --- |
| **(5’ to 3’)** |  |  |  |  |
| 19 | 0 | 0 | 0 | 0 |
| 20 | 0 | 0 | 0 | 0 |
| 21 | 0 | 0 | 0 | 0 |
| 22 | 0 | 0 | 0 | 0 |
| 23 | 0 | 0 | 0 | 0 |
| 24 | 0 | 0 | 0 | 0 |
| 25 | 0.0666 | 0.3333 | 0.4666 | 0.1333 |
| 26 | 0.6 | 0.1333 | 0 | 0.2666 |
| 27 | 0.2 | 0.4 | 0.2666 | 0.1333 |
| 28 | 0.2666 | 0.2 | 0.0666 | 0.4666 |
| 29 | 0 | 0.5333 | 0.4666 | 0 |
| 30 | 0.2666 | 0.4 | 0.2 | 0.1333 |
| 31 | 0 | 0.8666 | 0.1333 | 0 |
| 32 | 0 | 0 | 0 | 1 |
| 33 | 0.2666 | 0.5333 | 0.0666 | 0.1333 |
| 34 | 0.1333 | 0.2 | 0.1333 | 0.5333 |
| 35 | 1 | 0 | 0 | 0 |

Table S3: Predicted secondary structures of the substrates by mfold.

*The putative region (positions 20, 21, 22 and 23) on the substrates which might be critical for ligation by R18-T4, R18-T3, and R18-T2 ribozymes is shown with a line (curved or straight). This region in the group of substrates that were ligated is mostly unpaired and self-base paired in the ones not ligated.*

| Substrate  (5’ to 3’) | Predicted mfold structure | Substrate  (5’ to 3’) | Predicted mfold structure | Substrate  (5’ to 3’) | Predicted mfold structure | Substrate  (5’ to 3’) | Predicted mfold structure |
| --- | --- | --- | --- | --- | --- | --- | --- |
| 1 |  | 8A |  | G6 |  | 8B |  |
| 6 |  | G1 |  | G7 |  | 7B |  |
| 6A |  | G2 |  | 8 |  | 7A |  |
| 6B |  | G3 |  | 6C |  | 6E |  |
| 7 |  | G4 |  | 6D |  | G5 |  |
