# Supplementary material for: Molecular trade-offs in RNA ligases affected the modular emergence of complex ribozymes at the origin of life
Source: R Soc Open Sci. 2017 Sep 20;4(9):170376. doi: 10.1098/rsos.170376 (PMC5627087; doi:10.1098/rsos.170376)

**Section S1: Synthesis and Sequence details of R18 polymerase and its truncated molecules**

**
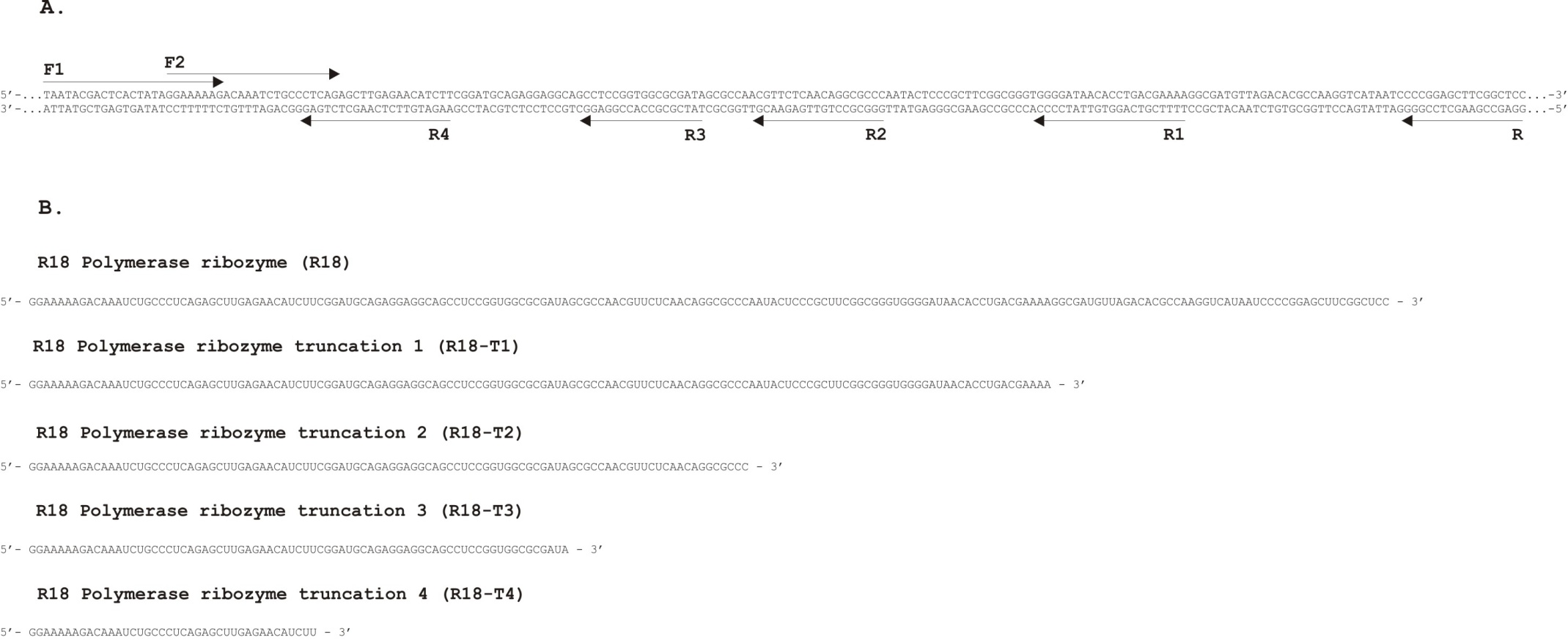
**

Figure S1: Synthesis of R18 polymerase (R18) and its truncated molecules

**A.** Template DNA sequence for R18; cloned and sequenced in pTZ57R/T vector (vector shown with dotted lines). The plasmid was used for amplification of template DNA sequences (using primers indicated by arrows). Primers F1 and R were used for amplification of template for R18, primers F1 and R1 were used for amplification of template for R18- T1, primers F1 and R2 were used for amplification of template for R18-T2, primers F1 and R3 were used for amplification of template for R18-T3, primers F1 and R4 were used for amplification of template for R18-T4. The templates were used for *in vitro* transcription of R18 and its truncated molecules. **B.**  RNA sequences of R18 and its truncations (shown in 5’ to 3’ direction) after *in vitro* transcription.

**Section S2. Schematic representation of self-ligation assay**


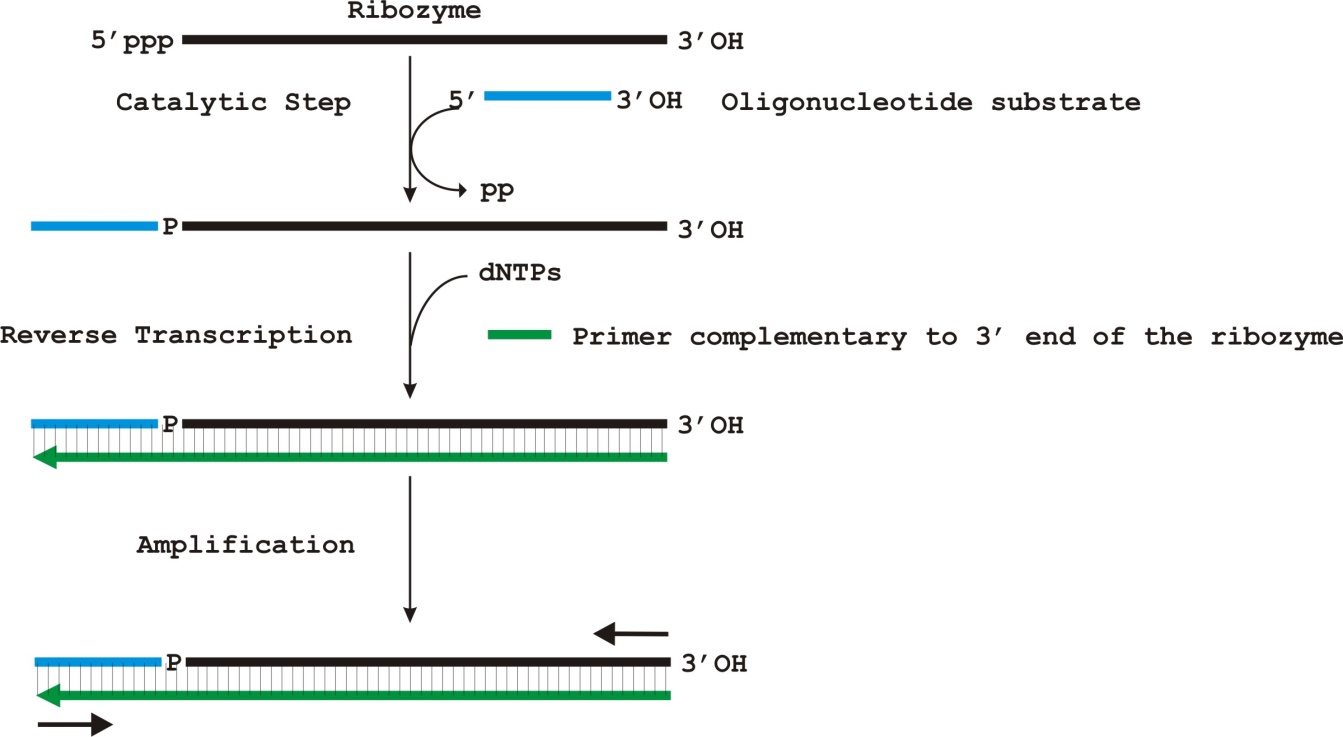


Figure S2: Schematic representation of the ribozyme self-ligation assay.

The black solid line represents the RNA (shown in 5’-3’ direction). The blue solid line represents the oligonucleotide substrate used in the assay (shown in 5’ - 3’ direction). The green solid arrow line represents the cDNA formed from the reverse transcription of the ligated product using the primer complementary to the 3’ end of the ribozyme. The primers used to amplify the reverse transcribed product are shown as black solid arrows.

**Section S3. Details of PCR conditions for detection of the self-ligation reactions**

Briefly, the PCR was carried out using *Pfu* DNA polymerase with 25 pmoles of each forward and reverse primers in a total volume of 50 µl. The following thermocycling conditions were used: Initial denaturation at 95 ºC for 5 mins, 30 cycles of [denaturation at 95 ºC for 1 min, annealing at 58 ºC for 30 sec, extension at 72 ºC for 30 sec], and final extension at 72 ºC for 10 mins. The schematic representation of the assay is shown in Figure below. The self-ligation activity of the RNA with the oligonucleotide substrate was assessed by comparing the size of the amplicon from the assay to the respective reference DNA. A 35bp difference in size was indicative of ligation reaction. The positive activity was further confirmed by sequence analysis of the PCR products.

**Section S4: Real-time quantitative PCR conditions and determination of rate of self-ligation activity of ribozymes with substrates**

### S4.1: PCR conditions of Real-time quantitative PCR of ribozyme assays

Real-time quantitative PCR was performed with the following thermal cycling conditions; Step 1: 50 ºC for 2 mins, Step 2: 95 ºC for 10 mins, Step 3: 95 ºC for 15 sec, Step 4: 60 ºC for 1 min. Step 3 and 4 were repeated for 40 cycles. Fluorescence was measured at 530 nm.

S4.2: Quantification of cDNA copies of ligated product formed in ribozyme assays

The CP values of known copies of DNA were obtained. The CP value corresponds to the cycle number at which there is first detectable increase in fluorescence as a result of cleavage of probe during polymerisation reaction (described in Figure S4.1 and S4.2). A standard curve was generated using base 10 log of initial target copy number versus corresponding CP value (given in Section SF). The standard curve was used to quantify unknown cDNA copies produced from the ribozyme reaction product at different time intervals. For an equivalent CP value, the number of cDNA copies present in the ribozyme reaction was quantified to be one half of the copies calculated from the standard curve sample (dsDNA). All the PCR assays included a control reaction with no DNA and each run included a sample of standard curve for calibration.


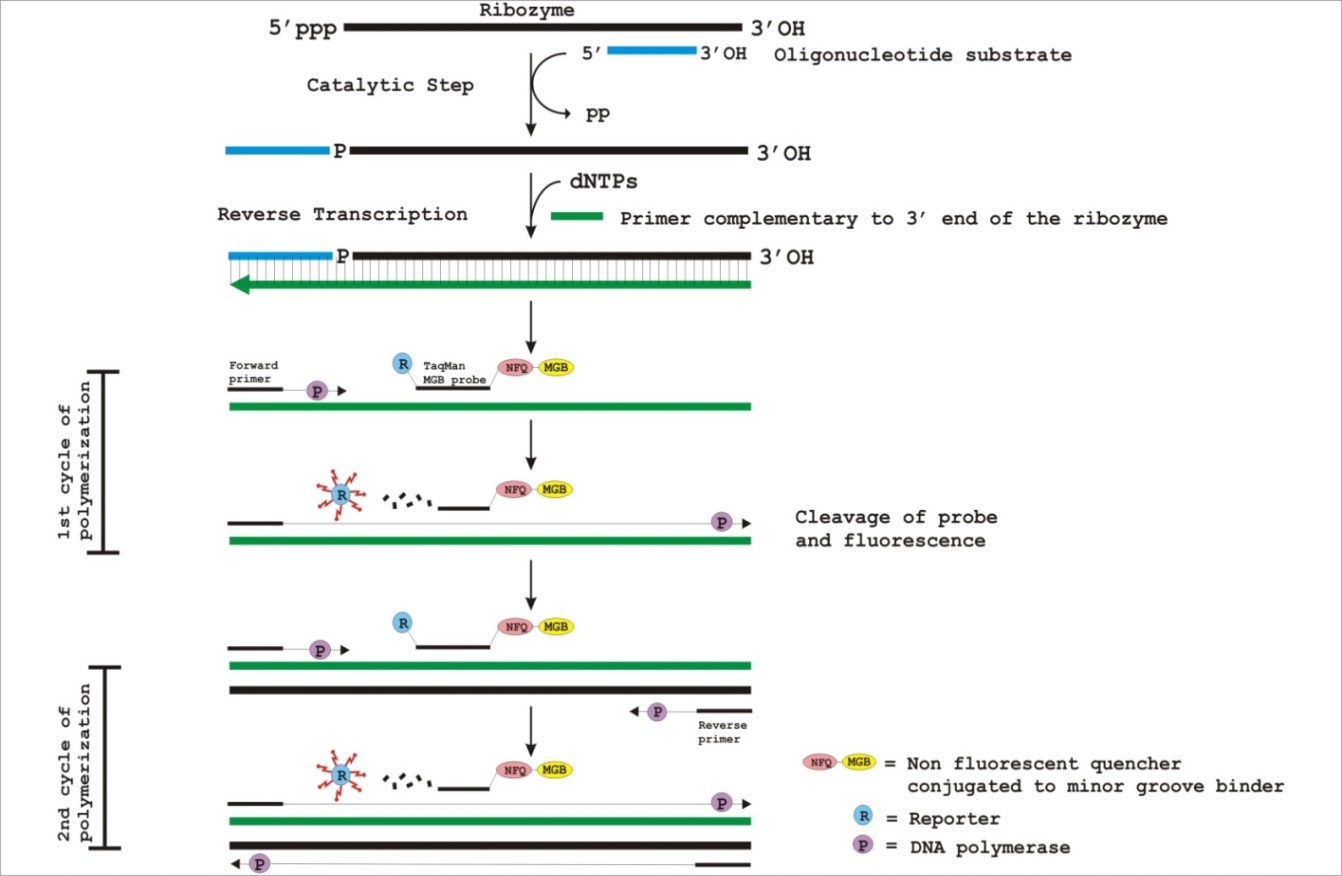


Figure S4.1: Schematic representation of the assay performed for quantitative analysis of the ribozyme’s ligation activity.

The ribozymes reaction products were reverse transcribed and PCR amplified using a probe designed to bind a specific region common in the reverse transcribed sequences of the ribozyme ligation product. When the probe is intact, signals from reporter dye FAM are quenched by NFQ. During the PCR reaction, the 5’ nuclease activity of DNA polymerase cleaves the probe separating FAM and NFQ, resulting in fluorescence of FAM.


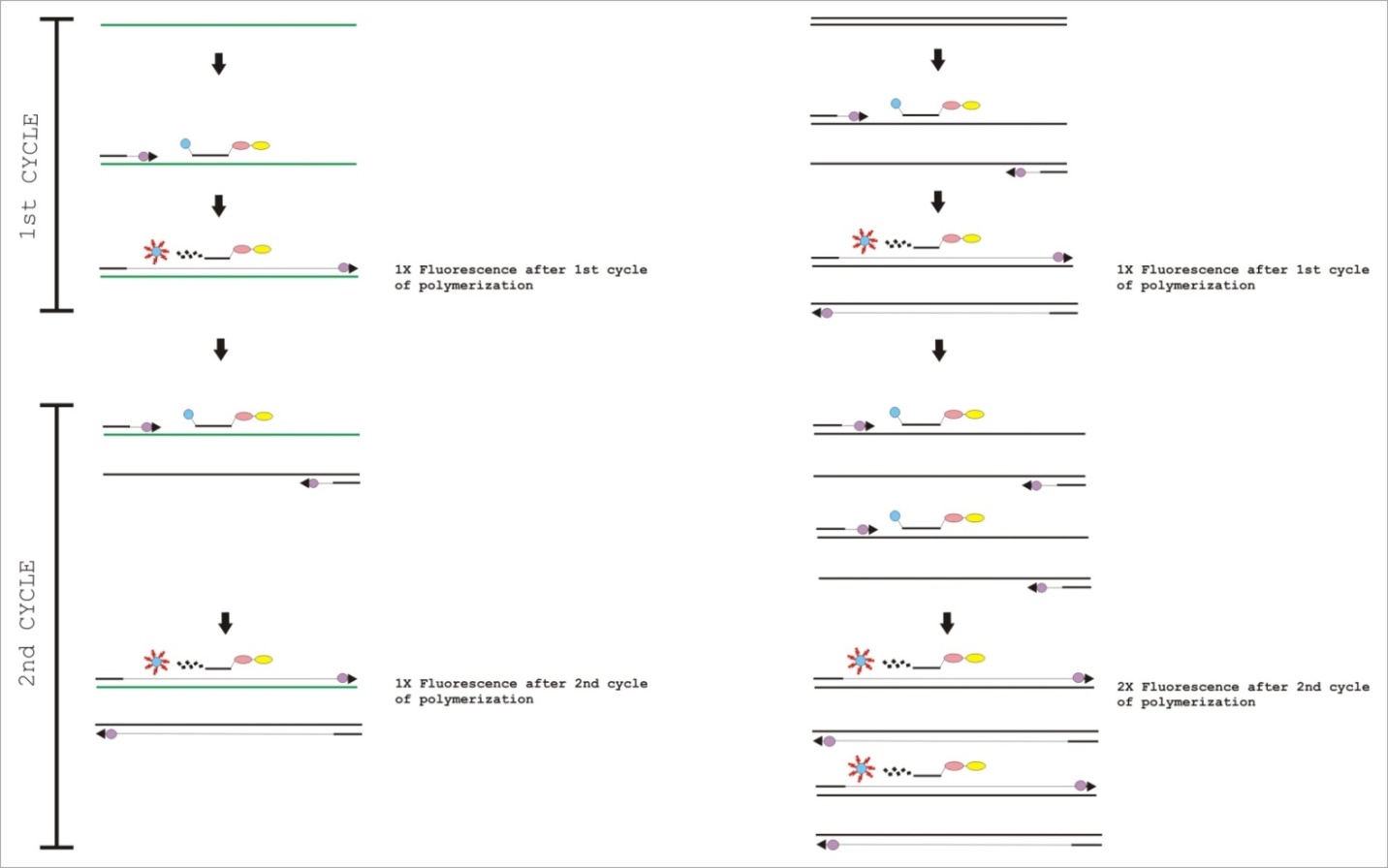


Figure S4.2: Comparison of the fluorescence from ribozyme self-ligation assay and the corresponding standard sample.

Quantitative comparison of the fluorescence after 2 cycles of polymerisation using real time PCR from cDNA of ribozyme’s self-ligation reaction (left) and the corresponding dsDNA used as standard (right).

**S4.3: Time course analysis of self-ligation activity of ribozymes**

A graph of cDNA copies quantified in the reaction was plotted versus duration of incubation using Microsoft Excel. The rate of reaction was determined from the slope of the curve and was given as the copies of ligated product cDNA formed per minute.

Figure S4.3: Time course analysis of R18-T4 ribozyme self-ligation activity.

The X axis shows the incubation time (in minutes). The Y axis shows the copies of cDNA formed in the reaction. The symbols represent the activity of the R18-T4 ribozyme with the substrates in the course of time.

Figure S4.4: Time course analysis of R18-T3 ribozyme self-ligation activity.

The X axis shows the incubation time (in minutes). The Y axis shows the copies of cDNA formed in the reaction. The symbols represent the activity of the R18-T3 ribozyme with the substrates in the course of time.

Figure S4.5: Time course analysis of R18-T2 ribozyme self-ligation activity.

The X axis shows the incubation time (in minutes). The Y axis shows the copies of cDNA formed in the reaction. The symbols represent the activity of the R18-T2 ribozyme with the substrates in the course of time.

Figure S4.6: Time course analysis of R18-T1 ribozyme self-ligation activity.

The X axis shows the incubation time (in minutes). The Y axis shows the copies of cDNA formed in the reaction. The symbols represent the activity of the R18-T1 ribozyme with the substrates in the course of time.

Figure S4.7: Time course analysis of R18 ribozyme self-ligation activity.

The X axis shows the incubation time (in minutes). The Y axis shows the copies of cDNA formed in the reaction. The symbols represent the activity of the R18 ribozyme with the substrates in the course of time.


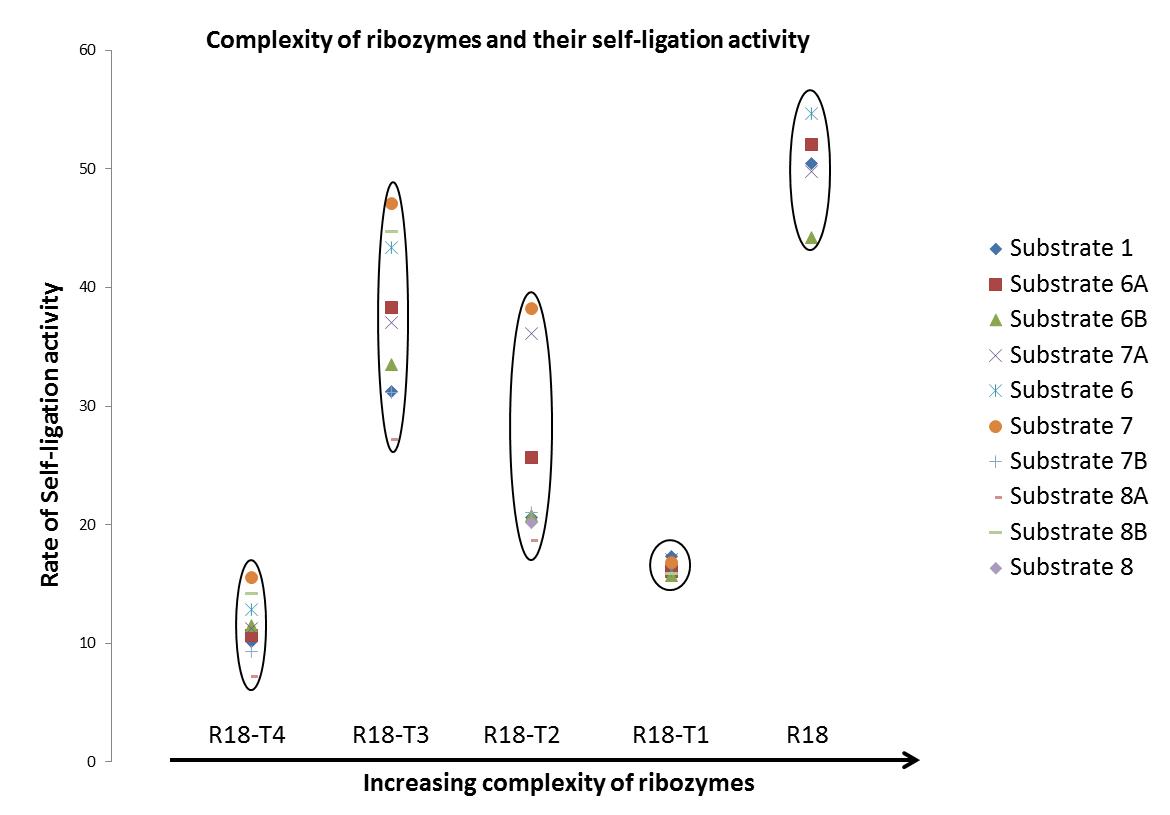


Figure S4.8: Consistency of the ribozymes with respect to their rate of self-ligation reaction with the substrates.

The X axis represents the increase in size and structural complexity of ribozymes. The Y axis represents the copies of the ligated product cDNA formed per minute. The circles denote the consistency of rate of ribozyme activity with different substrates.


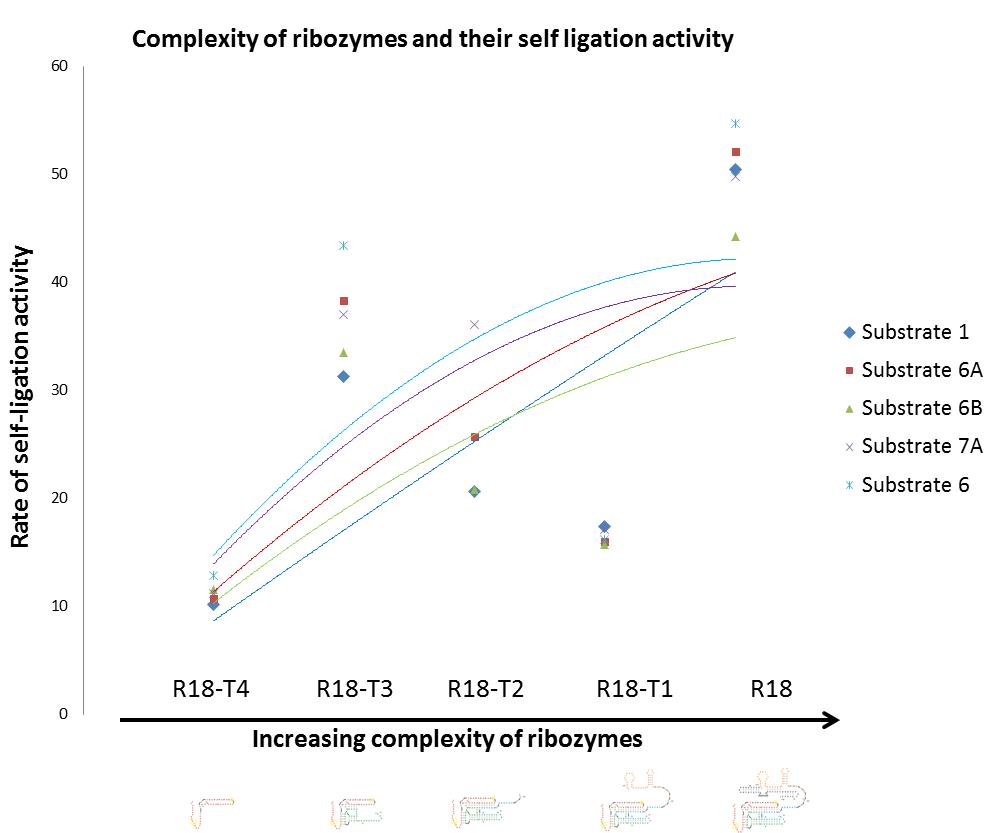


Figure S4.9: Correlation of complexity of the ribozymes with respect to their rate of self-ligation activity with the substrates.

The X axis represents the increase in size and structural complexity of ribozymes. The Y axis represents the copies of ligated product cDNA formed per minute (given in Table 3.13). The curves represent a linear transition in the efficiency of the ribozyme with increase in their size and structural complexity.

## Section SA: Confirmation of self-ligation activity of R18 RNAs by sequence analysis

The amplicons (224 bp in size) indicating self-ligation of R18 RNA with each of the substrates 1, 2, 3, 6, 6a, 6b, 7a were sequenced (top) and aligned with the expected sequence (below). Shown are sequence alignments of ligated product of R18 with (I) oligonucleotide substrate 1 (II) oligonucleotide substrate 2 (III) oligonucleotide substrate 3 (IV) oligonucleotide substrate 6 (V) oligonucleotide substrate 6a (VI) oligonucleotide substrate 6b (VII) oligonucleotide substrate 7a.

**(I)**

EMBOSS_001 51 CGGGCCCGGGATCCGATTCTCGACGTCAGCCTGGACTAATACGACTCACT 100

||||||||||||||||||||||||||||||||

EMBOSS_001 1 ------------------CTCGACGTCAGCCTGGACTAATACGACTCACT 32

EMBOSS_001 101 ATAGGAAAAAGACAAATCTGCCCTCAGAGCTTGAGAACATCTTCGGATGC 150

||||||||||||||||||||||||||||||||||||||||||||||||||

EMBOSS_001 33 ATAGGAAAAAGACAAATCTGCCCTCAGAGCTTGAGAACATCTTCGGATGC 82

EMBOSS_001 151 AGAGGAGGCAGCCTCCGGTGGCGCGATAGCGCCAACGTTCTCAACAGGCG 200

||||||||||||||||||||||||||||||||||||||||||||||||||

EMBOSS_001 83 AGAGGAGGCAGCCTCCGGTGGCGCGATAGCGCCAACGTTCTCAACAGGCG 132

EMBOSS_001 201 CCCAATACTCCCGCTTCGGCGGGTGGGGATAACACCTGACGAAAAGGCGA 250

||||||||||||||||||||||||||||||||||||||||||||||||||

EMBOSS_001 133 CCCAATACTCCCGCTTCGGCGGGTGGGGATAACACCTGACGAAAAGGCGA 182

EMBOSS_001 251 TGTTAGACACGCCAAGGTCATAATCCCCGGAGCTTCGGCTCCAATCTAGA 300

||||||||||||||||||||||||||||||||||||||||||

EMBOSS_001 183 TGTTAGACACGCCAAGGTCATAATCCCCGGAGCTTCGGCTCC-------- 224

**(II)**

EMBOSS_001 51 ACGGGCCCGGGATCCGATTGYCMACTTCCGCATGAACGAATACTACGCAC 100

|.|.|||||||||||||||||||||||||||

EMBOSS_001 1 -------------------GTCAACTTCCGCATGAACGAATACTACGCAC 31

EMBOSS_001 101 TAAAGGAAAAAGACAAATCTGCCCTCAGAGCTTGAGAACATCTTCGGATG 150

||||||||||||||||||||||||||||||||||||||||||||||||||

EMBOSS_001 32 TAAAGGAAAAAGACAAATCTGCCCTCAGAGCTTGAGAACATCTTCGGATG 81

EMBOSS_001 151 CAGAGGAGGCAGCCTCCGGTGGCGCGATAGCGCCAACGTTCTCAACAGGC 200

||||||||||||||||||||||||||||||||||||||||||||||||||

EMBOSS_001 82 CAGAGGAGGCAGCCTCCGGTGGCGCGATAGCGCCAACGTTCTCAACAGGC 131

EMBOSS_001 201 GCCCAATACTCCCGCTTCGGCGGGTGGGGATAACACCTGACGAAAAGGCG 250

||||||||||||||||||||||||||||||||||||||||||||||||||

EMBOSS_001 132 GCCCAATACTCCCGCTTCGGCGGGTGGGGATAACACCTGACGAAAAGGCG 181

EMBOSS_001 251 ATGTTAGACACGCCAAGGTCATAATCCCCGGAGCTTCGGCTCCAATCTAG 300

|||||||||||||||||||||||||||||||||||||||||||

EMBOSS_001 182 ATGTTAGACACGCCAAGGTCATAATCCCCGGAGCTTCGGCTCC------- 224

**(III)**

EMBOSS_001 51 GGGCCCGGGATCCGATTCACSACGACAACCTGGTCTAATACGCCTCACGA 100

|||.|||||||||||||||||||||||||||||

EMBOSS_001 1 -----------------CACGACGACAACCTGGTCTAATACGCCTCACGA 33

EMBOSS_001 101 TAGGAAAAAGACAAATCTGCCCTCAGAGCTTGAGAACATCTTCGGATGCA 150

||||||||||||||||||||||||||||||||||||||||||||||||||

EMBOSS_001 34 TAGGAAAAAGACAAATCTGCCCTCAGAGCTTGAGAACATCTTCGGATGCA 83

EMBOSS_001 151 GAGGAGGCAGCCTCCGGTGGCGCGATAGCGCCAACGTTCTCAACAGGCGC 200

||||||||||||||||||||||||||||||||||||||||||||||||||

EMBOSS_001 84 GAGGAGGCAGCCTCCGGTGGCGCGATAGCGCCAACGTTCTCAACAGGCGC 133

EMBOSS_001 201 CCAATACTCCCGCTTCGGCGGGTGGGGATAACACCTGACGAAAAGGCGAT 250

||||||||||||||||||||||||||||||||||||||||||||||||||

EMBOSS_001 134 CCAATACTCCCGCTTCGGCGGGTGGGGATAACACCTGACGAAAAGGCGAT 183

EMBOSS_001 251 GTTAGACACGCCAAGGTCATAATCCCCGGAGCTTCGGCTCCAATCTAGAT 300

|||||||||||||||||||||||||||||||||||||||||

EMBOSS_001 184 GTTAGACACGCCAAGGTCATAATCCCCGGAGCTTCGGCTCC--------- 224

**(IV)**

EMBOSS_001 851 CGAATGCATCTAGATTCGACGTCAGCCTGGACTAATACTAAAAACTATAG 900

.|||||||||||||||||||||||||||||||||||

EMBOSS_001 1 --------------CTCGACGTCAGCCTGGACTAATACTAAAAACTATAG 36

EMBOSS_001 901 GAAAAAGACAAATCTGCCCTCAGAGCTTGAGAACATCTTCGGATGCAGAG 950

||||||||||||||||||||||||||||||||||||||||||||||||||

EMBOSS_001 37 GAAAAAGACAAATCTGCCCTCAGAGCTTGAGAACATCTTCGGATGCAGAG 86

EMBOSS_001 951 GAGGCAGCCTCCGGTGGCGCGATAGCGCCAACGTTCTCAACAGGCGCCCA 1000

||||||||||||||||||||||||||||||||||||||||||||||||||

EMBOSS_001 87 GAGGCAGCCTCCGGTGGCGCGATAGCGCCAACGTTCTCAACAGGCGCCCA 136

EMBOSS_001 1001 ATACTCCCGCTTCGGCGGGTGGGGATAACACCTGACGAAAAGGCGATGTT 1050

||||||||||||||||||||||||||||||||||||||||||||||||||

EMBOSS_001 137 ATACTCCCGCTTCGGCGGGTGGGGATAACACCTGACGAAAAGGCGATGTT 186

EMBOSS_001 1051 AGACACGCCAAGGTCATAATCCCCGGAGCTTCGGCTCCATCGGATCCCGG 1100

||||||||||||||||||||||||||||||||||||||

EMBOSS_001 187 AGACACGCCAAGGTCATAATCCCCGGAGCTTCGGCTCC------------ 224

**(V)**

EMBOSS_001 851 GCGAATGCATCTAGATTCGACGTCAGCCTGGACTAATACTATTTACTATA 900

.||||||||||||||||||||||||||||||||||

EMBOSS_001 1 ---------------CTCGACGTCAGCCTGGACTAATACTATTTACTATA 35

EMBOSS_001 901 GGAAAAAGACAAATCTGCCCTCAGAGCTTGAGAACATCTTCGGATGCAGA 950

||||||||||||||||||||||||||||||||||||||||||||||||||

EMBOSS_001 36 GGAAAAAGACAAATCTGCCCTCAGAGCTTGAGAACATCTTCGGATGCAGA 85

EMBOSS_001 951 GGAGGCAGCCTCCGGTGGCGCGATAGCGCCAACGTTCTCAACAGGCGCCC 1000

||||||||||||||||||||||||||||||||||||||||||||||||||

EMBOSS_001 86 GGAGGCAGCCTCCGGTGGCGCGATAGCGCCAACGTTCTCAACAGGCGCCC 135

EMBOSS_001 1001 AATACTCCCGCTTCGGCGGGTGGGGATAACACCTGACGAAAAGGCGATGT 1050

||||||||||||||||||||||||||||||||||||||||||||||||||

EMBOSS_001 136 AATACTCCCGCTTCGGCGGGTGGGGATAACACCTGACGAAAAGGCGATGT 185

EMBOSS_001 1051 TAGACACGCCAAGGTCATAATCCCCGGAGCTTCGGCTCCATCGGATCCCG 1100

|||||||||||||||||||||||||||||||||||||||

EMBOSS_001 186 TAGACACGCCAAGGTCATAATCCCCGGAGCTTCGGCTCC----------- 224

**(VI)**

EMBOSS_001 801 GACGGCCAGTGAATTCGAGCTCGGTACCTCGCGAATGCATCTAGATTCTC 850

|||

EMBOSS_001 1 -----------------------------------------------CTC 3

EMBOSS_001 851 GACGTCAGCCTGGACTAATACTAGGGACTATAGGAAAAAGACAAATCTGC 900

||||||||||||||||||||||||||||||||||||||||||||||||||

EMBOSS_001 4 GACGTCAGCCTGGACTAATACTAGGGACTATAGGAAAAAGACAAATCTGC 53

EMBOSS_001 901 CCTCAGAGCTTGAGAACATCTTCGGATGCAGAGGAGGCAGCCTCCGGTGG 950

||||||||||||||||||||||||||||||||||||||||||||||||||

EMBOSS_001 54 CCTCAGAGCTTGAGAACATCTTCGGATGCAGAGGAGGCAGCCTCCGGTGG 103

EMBOSS_001 951 CGCGATAGCGCCAACGTTCTCAACAGGCGCCCAATACTCCCGCTTCGGCG 1000

||||||||||||||||||||||||||||||||||||||||||||||||||

EMBOSS_001 104 CGCGATAGCGCCAACGTTCTCAACAGGCGCCCAATACTCCCGCTTCGGCG 153

EMBOSS_001 1001 GGTGGGGATAACACCTGACGAAAAGGCGATGTTAGACACGCCAAGGTCAT 1050

||||||||||||||||||||||||||||||||||||||||||||||||||

EMBOSS_001 154 GGTGGGGATAACACCTGACGAAAAGGCGATGTTAGACACGCCAAGGTCAT 203

EMBOSS_001 1051 AATCCCCGGAGCTTCGGCTATCGGATCCCGGGCCCGTCGACWGCRGAGGC 1100

|||||||||||||||||| |||

EMBOSS_001 204 AATCCCCGGAGCTTCGGC-------TCC---------------------- 224

**(VII)**

EMBOSS_001 51 CGACGGGCCCGGGATCCGATTCGACGTCAGCCTGGACTATATGGACTCAC 100

.||||||||||||||||||||||||||||||

EMBOSS_001 1 -------------------CTCGACGTCAGCCTGGACTATATGGACTCAC 31

EMBOSS_001 101 TATAGGAAAAAGACAAATCTGCCCTCAGAGCTTGAGAACATCTTCGGATG 150

||||||||||||||||||||||||||||||||||||||||||||||||||

EMBOSS_001 32 TATAGGAAAAAGACAAATCTGCCCTCAGAGCTTGAGAACATCTTCGGATG 81

EMBOSS_001 151 CAGAGGAGGCAGCCTCCGGTGGCGCGATAGCGCCAACGTTCTCAACAGGC 200

||||||||||||||||||||||||||||||||||||||||||||||||||

EMBOSS_001 82 CAGAGGAGGCAGCCTCCGGTGGCGCGATAGCGCCAACGTTCTCAACAGGC 131

EMBOSS_001 201 GCCCAATACTCCCGCTTCGGCGGGTGGGGATAACACCTGACGAAAAGGCG 250

||||||||||||||||||||||||||||||||||||||||||||||||||

EMBOSS_001 132 GCCCAATACTCCCGCTTCGGCGGGTGGGGATAACACCTGACGAAAAGGCG 181

EMBOSS_001 251 ATGTTAGACACGCCAAGGTCATAATCCCCGGAGCTTCGGCCATCTAGATG 300

|||||||||||||||||||||||||||||||||||||||| ||.

EMBOSS_001 182 ATGTTAGACACGCCAAGGTCATAATCCCCGGAGCTTCGGC--TCC----- 224

## Section SB: Confirmation of self-ligation activity of R18-T1 RNAs by sequence analysis

The amplicons (177 bp in size) indicating self-ligation activity of R18-T1 RNA with each of the substrates 1, 2, 3, 4, 6, 7, 6a, 6b, 7a, 8b were sequenced (top) and aligned with the expected sequence (below). Shown are sequence alignments of ligated product of R18-T1 with (I) oligonucleotide substrate 1 (II) oligonucleotide substrate 2 (III) oligonucleotide substrate 3 (IV) oligonucleotide substrate 4 (V) oligonucleotide substrate 6 (VI) oligonucleotide substrate 7 (VII) oligonucleotide substrate 6a (VIII) oligonucleotide substrate 6b (IX) oligonucleotide substrate 7a (X) oligonucleotide substrate 8b.

**(I)**

EMBOSS_001 901 CGGCCAGTGAATTCGAGCTCGGTACCTCGCGAATGCATCTAGATTCTCGA 950

|||||

EMBOSS_001 1 ---------------------------------------------CTCGA 5

EMBOSS_001 951 CGTCAGCCTGGACTAATACGACTCACTATAGGAAAAAGACAAATCTGCCC 1000

||||||||||||||||||||||||||||||||||||||||||||||||||

EMBOSS_001 6 CGTCAGCCTGGACTAATACGACTCACTATAGGAAAAAGACAAATCTGCCC 55

EMBOSS_001 1001 TCAGAGCTTGAGAACATCTTCGGATGCAGAGGAGGCAGCCTCCGGTGGCG 1050

||||||||||||||||||||||||||||||||||||||||||||||||||

EMBOSS_001 56 TCAGAGCTTGAGAACATCTTCGGATGCAGAGGAGGCAGCCTCCGGTGGCG 105

EMBOSS_001 1051 CGATAGCGCCAACGTTCTCAACAGGCGCCCAATACTCCCGCTTCGGCGGG 1100

||||||||||||||||||||||||||||||||||||||||||||||||||

EMBOSS_001 106 CGATAGCGCCAACGTTCTCAACAGGCGCCCAATACTCCCGCTTCGGCGGG 155

EMBOSS_001 1101 TGGGGATAACACCTGRCGAATCGGATCCCGGGCCCGTCGACTGCRGRGGC 1150

|||||||||||||||.||||..

EMBOSS_001 156 TGGGGATAACACCTGACGAAAA---------------------------- 177

**(II)**

EMBOSS_001 901 GCATCTAGATTGTCAACTTCCGCATGAACGAATACTACGCACTAAAGGAA 950

|||||||||||||||||||||||||||||||||||||||

EMBOSS_001 1 -----------GTCAACTTCCGCATGAACGAATACTACGCACTAAAGGAA 39

EMBOSS_001 951 AAAGACAAATCTGCCCTCAGAGCTTGAGAACATCTTCGGATGCAGAGGAG 1000

||||||||||||||||||||||||||||||||||||||||||||||||||

EMBOSS_001 40 AAAGACAAATCTGCCCTCAGAGCTTGAGAACATCTTCGGATGCAGAGGAG 89

EMBOSS_001 1001 GCAGCCTCCGGTGGCGCGATAGCGCCAACGTTCTCAACAGGCGCCCAATA 1050

||||||||||||||||||||||||||||||||||||||||||||||||||

EMBOSS_001 90 GCAGCCTCCGGTGGCGCGATAGCGCCAACGTTCTCAACAGGCGCCCAATA 139

EMBOSS_001 1051 CTCCCGCTTCGGCGGGTGGGGATAACACCTGACRAAAAAATCGGATCCCG 1100

|||||||||||||||||||||||||||||||||.||||

EMBOSS_001 140 CTCCCGCTTCGGCGGGTGGGGATAACACCTGACGAAAA------------ 177

**(III)**

EMBOSS_001 51 ACGGGCCCGGGATCCGATTCACGACGACAACCTGGTCTAATACGCCTCAC 100

|||||||||||||||||||||||||||||||

EMBOSS_001 1 -------------------CACGACGACAACCTGGTCTAATACGCCTCAC 31

EMBOSS_001 101 GATAGGAAAAAGACAAATCTGCCCTCAGAGCTTGAGAACATCTTCGGATG 150

||||||||||||||||||||||||||||||||||||||||||||||||||

EMBOSS_001 32 GATAGGAAAAAGACAAATCTGCCCTCAGAGCTTGAGAACATCTTCGGATG 81

EMBOSS_001 151 CAGAGGAGGCAGCCTCCGGTGGCGCGATAGCGCCAACGTTCTCAACAGGC 200

||||||||||||||||||||||||||||||||||||||||||||||||||

EMBOSS_001 82 CAGAGGAGGCAGCCTCCGGTGGCGCGATAGCGCCAACGTTCTCAACAGGC 131

EMBOSS_001 201 GCCCAATACTCCCGCTTCGGCGGGTGGGGATAACACCTGACGAAAAAATC 250

||||||||||||||||||||||||||||||||||||||||||||||

EMBOSS_001 132 GCCCAATACTCCCGCTTCGGCGGGTGGGGATAACACCTGACGAAAA---- 177

**(IV)**

EMBOSS_001 851 GGTACCTCGCGAATGCATCTAGATTCTGGATGTAAGTCTTGAATATATGG 900

|||||||||||||||||||||||||

EMBOSS_001 1 -------------------------CTGGATGTAAGTCTTGAATATATGG 25

EMBOSS_001 901 AATCGCTCGAGGAAAAAGACAAATCTGCCCTCAGAGCTTGAGAACATCTT 950

||||||||||||||||||||||||||||||||||||||||||||||||||

EMBOSS_001 26 AATCGCTCGAGGAAAAAGACAAATCTGCCCTCAGAGCTTGAGAACATCTT 75

EMBOSS_001 951 CGGATGCAGAGGAGGCAGCCTCCGGTGGCGCGATAGCGCCAACGTTCTCA 1000

||||||||||||||||||||||||||||||||||||||||||||||||||

EMBOSS_001 76 CGGATGCAGAGGAGGCAGCCTCCGGTGGCGCGATAGCGCCAACGTTCTCA 125

EMBOSS_001 1001 ACAGGCGCCCAATACTCCCGCTTCGGCGGGTGGGGATAACMCCTGACGAA 1050

||||||||||||||||||||||||||||||||||||||||.|||||||||

EMBOSS_001 126 ACAGGCGCCCAATACTCCCGCTTCGGCGGGTGGGGATAACACCTGACGAA 175

EMBOSS_001 1051 AAAATCGGATCCCGGGCCCGTCGACTGCAGAGGCCTGCATGCAAGCTTCC 1100

||

EMBOSS_001 176 AA------------------------------------------------ 177

**(V)**

EMBOSS_001 851 CCAGTGAATTCGAGCTCGGTACCTCGCGAATGCATCTAGATTCTCGACGT 900

||||||||

EMBOSS_001 1 ------------------------------------------CTCGACGT 8

EMBOSS_001 901 CAGCCTGGACTAATACTAAAAACTATAGGAAAAAGACAAATCTGCCCTCA 950

||||||||||||||||||||||||||||||||||||||||||||||||||

EMBOSS_001 9 CAGCCTGGACTAATACTAAAAACTATAGGAAAAAGACAAATCTGCCCTCA 58

EMBOSS_001 951 GAGCTTGAGAACATCTTCGGATGCAGAGGAGGCAGCCTCCGGTGGCGCGA 1000

||||||||||||||||||||||||||||||||||||||||||||||||||

EMBOSS_001 59 GAGCTTGAGAACATCTTCGGATGCAGAGGAGGCAGCCTCCGGTGGCGCGA 108

EMBOSS_001 1001 TAGCGCCAACGTTCTCAACAGGCGCCCAATACTCCCGCTTCGGCGGGTGG 1050

||||||||||||||||||||||||||||||||||||||||||||||||||

EMBOSS_001 109 TAGCGCCAACGTTCTCAACAGGCGCCCAATACTCCCGCTTCGGCGGGTGG 158

EMBOSS_001 1051 GGATAACACCTGACGAAAAATCGGATCCCGGGCCCGTCGACTGCRGAGGC 1100

|||||||||||||||||||

EMBOSS_001 159 GGATAACACCTGACGAAAA------------------------------- 177

**(VI)**

EMBOSS_001 51 GACGGGCCCGGGATCCGATTCSACGTCAGCCTGGACTAATACTATTTACT 100

.||.||||||||||||||||||||||||||||

EMBOSS_001 1 ------------------CTCGACGTCAGCCTGGACTAATACTATTTACT 32

EMBOSS_001 101 ATAGGAAAAAGACAAATCTGCCCTCAGAGCTTGAGAACATCTTCGGATGC 150

||||||||||||||||||||||||||||||||||||||||||||||||||

EMBOSS_001 33 ATAGGAAAAAGACAAATCTGCCCTCAGAGCTTGAGAACATCTTCGGATGC 82

EMBOSS_001 151 AGAGGAGGCAGCCTCCGGTGGCGCGATAGCGCCAACGTTCTCAACAGGCG 200

||||||||||||||||||||||||||||||||||||||||||||||||||

EMBOSS_001 83 AGAGGAGGCAGCCTCCGGTGGCGCGATAGCGCCAACGTTCTCAACAGGCG 132

EMBOSS_001 201 CCCAATACTCCCGCTTCGGCGGGTGGGGATAACACCTGACGAAAATCTAG 250

|||||||||||||||||||||||||||||||||||||||||||||

EMBOSS_001 133 CCCAATACTCCCGCTTCGGCGGGTGGGGATAACACCTGACGAAAA----- 177

**(VII)**

EMBOSS_001 51 AGATTCTCGACGTCAGCCTGGACTAATACTAGGGACTATAGGAAAAAGAC 100

|||||||||||||||||||||||||||||||||||||||||||||

EMBOSS_001 1 -----CTCGACGTCAGCCTGGACTAATACTAGGGACTATAGGAAAAAGAC 45

EMBOSS_001 101 AAATCTGCCCTCAGAGCTTGAGAACATCTTCGGATGCAGAGGAGGCAGCC 150

||||||||||||||||||||||||||||||||||||||||||||||||||

EMBOSS_001 46 AAATCTGCCCTCAGAGCTTGAGAACATCTTCGGATGCAGAGGAGGCAGCC 95

EMBOSS_001 151 TCCGGTGGCGCGATAGCGCCAACGTTCTCAACAGGCGCCCAATACTCCCG 200

||||||||||||||||||||||||||||||||||||||||||||||||||

EMBOSS_001 96 TCCGGTGGCGCGATAGCGCCAACGTTCTCAACAGGCGCCCAATACTCCCG 145

EMBOSS_001 201 CTTCGGCGGGTGGGGATAACACCTGACGAAAAAATCGGATCCCGGGCCCG 250

||||||||||||||||||||||||||||||||

EMBOSS_001 146 CTTCGGCGGGTGGGGATAACACCTGACGAAAA------------------ 177

**(VIII)**

EMBOSS_001 901 TAGATTCTCGACGTCAGCCTGGACTATATGGACTCACTATAGGAAAAAGA 950

||||||||||||||||||||||||||||||||||||||||||||

EMBOSS_001 1 ------CTCGACGTCAGCCTGGACTATATGGACTCACTATAGGAAAAAGA 44

EMBOSS_001 951 CAAATCTGCCCTCAGAGCTTGAGAACATCTTCGGATGCAGAGGAGGCAGC 1000

||||||||||||||||||||||||||||||||||||||||||||||||||

EMBOSS_001 45 CAAATCTGCCCTCAGAGCTTGAGAACATCTTCGGATGCAGAGGAGGCAGC 94

EMBOSS_001 1001 CTCCGGTGGCGCGATAGCGCCAACGTTCTCAACAGGCGCCCAATACTCCC 1050

||||||||||||||||||||||||||||||||||||||||||||||||||

EMBOSS_001 95 CTCCGGTGGCGCGATAGCGCCAACGTTCTCAACAGGCGCCCAATACTCCC 144

EMBOSS_001 1051 GCTTCGGCGGGTGGGGATAACACCTRACGAAAAAATCGGATCCCGGGCCC 1100

|||||||||||||||||||||||||.|||||||

EMBOSS_001 145 GCTTCGGCGGGTGGGGATAACACCTGACGAAAA----------------- 177

**(IX)**

EMBOSS_001 901 CGAATGCATCTAGATTCTCGACGTCAGCCTGGACTATATGGAATCGCTCG 950

||||||||||||||||||||||||||||||||||

EMBOSS_001 1 ----------------CTCGACGTCAGCCTGGACTATATGGAATCGCTCG 34

EMBOSS_001 951 AGGAAAAAGACAAATCTGCCCTCAGAGCTTGAGAACATCTTCGGATGCAG 1000

||||||||||||||||||||||||||||||||||||||||||||||||||

EMBOSS_001 35 AGGAAAAAGACAAATCTGCCCTCAGAGCTTGAGAACATCTTCGGATGCAG 84

EMBOSS_001 1001 AGGAGGCAGCCTCCGGTGGCGCGATAGCGCCAACGTTCTCAACAGGCGCC 1050

||||||||||||||||||||||||||||||||||||||||||||||||||

EMBOSS_001 85 AGGAGGCAGCCTCCGGTGGCGCGATAGCGCCAACGTTCTCAACAGGCGCC 134

EMBOSS_001 1051 CAATACTCCCGCTTCGGCGGGTGGGGATAACACCTGACGAAAAAATCGGA 1100

|||||||||||||||||||||||||||||||||||||||||||

EMBOSS_001 135 CAATACTCCCGCTTCGGCGGGTGGGGATAACACCTGACGAAAA------- 177

**(X)**

EMBOSS_001 1 ------CTCGACGTCAGCCTGGACTAATACCCTCGCCTCAAGGAAAAAGA 44

||||||||||||||||||||||||||||||||||||||||||||

EMBOSS_001 901 TAGATTCTCGACGTCAGCCTGGACTAATACCCTCGCCTCAAGGAAAAAGA 950

EMBOSS_001 45 CAAATCTGCCCTCAGAGCTTGAGAACATCTTCGGATGCAGAGGAGGCAGC 94

||||||||||||||||||||||||||||||||||||||||||||||||||

EMBOSS_001 951 CAAATCTGCCCTCAGAGCTTGAGAACATCTTCGGATGCAGAGGAGGCAGC 1000

EMBOSS_001 95 CTCCGGTGGCGCGATAGCGCCAACGTTCTCAACAGGCGCCCAATACTCCC 144

||||||||||||||||||||||||||||||||||||||||||||||||||

EMBOSS_001 1001 CTCCGGTGGCGCGATAGCGCCAACGTTCTCAACAGGCGCCCAATACTCCC 1050

EMBOSS_001 145 GCTTCGGCGGGTGGGGATAACACCTGACGAAAA----------------- 177

|||||||||||||||||||||.|||||||||||

EMBOSS_001 1051 GCTTCGGCGGGTGGGGATAACWCCTGACGAAAAAATCGGATCCCGGGCCC 1100

## Section SC: Confirmation of self-ligation activity of R18-T2 RNAs by sequence analysis

The amplicons (135 bp in size) indicating self-ligation activity of R18-T2 RNA with each of the substrates 1, 2, 3, 4, 5, 7, 8, 6a, 6b, 7a, 7b, 8a were sequenced (top) and aligned with the expected sequence (below). Shown are sequence alignments of ligated product of R18-T2 with (I) oligonucleotide substrate 1 (II) oligonucleotide substrate 2 (III) oligonucleotide substrate 3 (IV) oligonucleotide substrate 4 (V) oligonucleotide substrate 5 (VI) oligonucleotide substrate 7 (VII) oligonucleotide substrate 8 (VIII) oligonucleotide substrate 6a (IX) oligonucleotide substrate 6b (X) oligonucleotide substrate 7a (XI) oligonucleotide substrate 7b (XII) oligonucleotide substrate 8a

**(I)**

EMBOSS_001 951 ATTCTCGACGTCAGCCTGGACTAATACGACTCACTATAGGAAAAAGACAA 1000

|||||||||||||||||||||||||||||||||||||||||||||||

EMBOSS_001 1 ---CTCGACGTCAGCCTGGACTAATACGACTCACTATAGGAAAAAGACAA 47

EMBOSS_001 1001 ATCTGCCCTCAGAGCTTGAGAACATCTTCGGATGCAGAGGAGGCAGCCTC 1050

||||||||||||||||||||||||||||||||||||||||||||||||||

EMBOSS_001 48 ATCTGCCCTCAGAGCTTGAGAACATCTTCGGATGCAGAGGAGGCAGCCTC 97

EMBOSS_001 1051 CGGTGGCGCGATAGCGCCAACGTTCTCAACAGGCGSCCAATCGGATCCCG 1100

|||||||||||||||||||||||||||||||||||.||

EMBOSS_001 98 CGGTGGCGCGATAGCGCCAACGTTCTCAACAGGCGCCC------------ 135

**(II)**

EMBOSS_001 951 TCTAGATTGTCAACTTCCGCATGAACGAATACTACGCACTAAAGGAAAAA 1000

||||||||||||||||||||||||||||||||||||||||||

EMBOSS_001 1 --------GTCAACTTCCGCATGAACGAATACTACGCACTAAAGGAAAAA 42

EMBOSS_001 1001 GACAAATCTGCCCTCAGAGCTTGAGAACATCTTCGGATGCAGAGGAGGCA 1050

||||||||||||||||||||||||||||||||||||||||||||||||||

EMBOSS_001 43 GACAAATCTGCCCTCAGAGCTTGAGAACATCTTCGGATGCAGAGGAGGCA 92

EMBOSS_001 1051 GCCTCCGGTGGCGCGATAGCGCCAACGTTCTCAACAGGCGCSCAATCGGA 1100

|||||||||||||||||||||||||||||||||||||||||.|

EMBOSS_001 93 GCCTCCGGTGGCGCGATAGCGCCAACGTTCTCAACAGGCGCCC------- 135

**(III)**

EMBOSS_001 51 ACGGGCCCGGGATCCGATTCACGACGACAACCTGGTCTAATACGCCTCAC 100

|||||||||||||||||||||||||||||||

EMBOSS_001 1 -------------------CACGACGACAACCTGGTCTAATACGCCTCAC 31

EMBOSS_001 101 GATAGGAAAAAGACAAATCTGCCCTCAGAGCTTGAGAACATCTTCGGATG 150

||||||||||||||||||||||||||||||||||||||||||||||||||

EMBOSS_001 32 GATAGGAAAAAGACAAATCTGCCCTCAGAGCTTGAGAACATCTTCGGATG 81

EMBOSS_001 151 CAGAGGAGGCAGCCTCCGGTGGCGCGATAGCGCCAACGTTCTCAACAGGC 200

||||||||||||||||||||||||||||||||||||||||||||||||||

EMBOSS_001 82 CAGAGGAGGCAGCCTCCGGTGGCGCGATAGCGCCAACGTTCTCAACAGGC 131

EMBOSS_001 201 GCCCAATCTAGATGCATTCGCGAGGTACCGAGCTCGAATTCACTGGCCGT 250

||||

EMBOSS_001 132 GCCC---------------------------------------------- 135

**(IV)**

EMBOSS_001 951 TCGGTACCTCGCGAATGCATCTAGATTCTGGATGTAAGTCTTGAATATAT 1000

|||||||||||||||||||||||

EMBOSS_001 1 ---------------------------CTGGATGTAAGTCTTGAATATAT 23

EMBOSS_001 1001 GGAATCGCTCGAGGAAAAAGACAAATCTGCCCTCAGAGCTTGAGAACATC 1050

||||||||||||||||||||||||||||||||||||||||||||||||||

EMBOSS_001 24 GGAATCGCTCGAGGAAAAAGACAAATCTGCCCTCAGAGCTTGAGAACATC 73

EMBOSS_001 1051 TTCGGATGCAGAGGAGGCAGCCTCCGGTGGCGCGATAGCGCCAACGTTCT 1100

||||||||||||||||||||||||||||||||||||||||||||||||||

EMBOSS_001 74 TTCGGATGCAGAGGAGGCAGCCTCCGGTGGCGCGATAGCGCCAACGTTCT 123

EMBOSS_001 1101 CWACAGGCGCCCAATCGGATCCCGGGCCCGTCGACTGCAGAGGCCTGCAT 1150

|.||||||||||

EMBOSS_001 124 CAACAGGCGCCC-------------------------------------- 135

**(V)**

EMBOSS_001 51 GACGGGCCCGGGATCCGATTTAATACTCATAACGACTACATGGACCTCGC 100

||||||||||||||||||||||||||||||

EMBOSS_001 1 --------------------TAATACTCATAACGACTACATGGACCTCGC 30

EMBOSS_001 101 CTCAAGGAAAAAGACAAATCTGCCCTCAGAGCTTGAGAACATCTTCGGAT 150

||||||||||||||||||||||||||||||||||||||||||||||||||

EMBOSS_001 31 CTCAAGGAAAAAGACAAATCTGCCCTCAGAGCTTGAGAACATCTTCGGAT 80

EMBOSS_001 151 GCAGAGGAGGCAGCCTCCGGTGGCGCGATAGCGCCAACGTTCTCAACAGG 200

||||||||||||||||||||||||||||||||||||||||||||||||||

EMBOSS_001 81 GCAGAGGAGGCAGCCTCCGGTGGCGCGATAGCGCCAACGTTCTCAACAGG 130

EMBOSS_001 201 CGCCCAATCTAGATGCATTCGCGAGGTACCGAGCTCGAATTCACTGGCCG 250

|||||

EMBOSS_001 131 CGCCC--------------------------------------------- 135

**(VI)**

EMBOSS_001 951 CTCGACGTCAGCCTGGACTAATACTATTTACTATAGGAAAAAGACAAATC 1000

||||||||||||||||||||||||||||||||||||||||||||||||||

EMBOSS_001 1 CTCGACGTCAGCCTGGACTAATACTATTTACTATAGGAAAAAGACAAATC 50

EMBOSS_001 1001 TGCCCTCAGAGCTTGAGAACATCTTCGGATGCAGAGGAGGCAGCCTCCGG 1050

||||||||||||||||||||||||||||||||||||||||||||||||||

EMBOSS_001 51 TGCCCTCAGAGCTTGAGAACATCTTCGGATGCAGAGGAGGCAGCCTCCGG 100

EMBOSS_001 1051 TGGCGCGATAGCGCCAACGTTCTCAACAGGCGCCAATCGGATCCCGGGCC 1100

||||||||||||||||||||||||||||||||||.

EMBOSS_001 101 TGGCGCGATAGCGCCAACGTTCTCAACAGGCGCCC--------------- 135

**(VII)**

EMBOSS_001 51 AGATTCTCGACGTCAGCCTGGACTAATACTAGGGACTATAGGAAAAAGAC 100

|||||||||||||||||||||||||||||||||||||||||||||

EMBOSS_001 1 -----CTCGACGTCAGCCTGGACTAATACTAGGGACTATAGGAAAAAGAC 45

EMBOSS_001 101 AAATCTGCCCTCAGAGCTTGAGAACATCTTCGGATGCAGAGGAGGCAGCC 150

||||||||||||||||||||||||||||||||||||||||||||||||||

EMBOSS_001 46 AAATCTGCCCTCAGAGCTTGAGAACATCTTCGGATGCAGAGGAGGCAGCC 95

EMBOSS_001 151 TCCGGTGGCGCGATAGCGCCAACGTTCTCAACAGGCGCCCAATCGGATCC 200

||||||||||||||||||||||||||||||||||||||||

EMBOSS_001 96 TCCGGTGGCGCGATAGCGCCAACGTTCTCAACAGGCGCCC---------- 135

**(VIII)**

EMBOSS_001 951 ATGCATCTAGATTCTCGACGTCAGCCTGGACTATATGGACTCACTATAGG 1000

|||||||||||||||||||||||||||||||||||||

EMBOSS_001 1 -------------CTCGACGTCAGCCTGGACTATATGGACTCACTATAGG 37

EMBOSS_001 1001 AAAAAGACAAATCTGCCCTCAGAGCTTGAGAACATCTTCGGATGCAGAGG 1050

||||||||||||||||||||||||||||||||||||||||||||||||||

EMBOSS_001 38 AAAAAGACAAATCTGCCCTCAGAGCTTGAGAACATCTTCGGATGCAGAGG 87

EMBOSS_001 1051 AGGCAGCCTCCGGTGGCGCGATAGCGCCAACGTTCTCAACAGGSGCCCAA 1100

|||||||||||||||||||||||||||||||||||||||||||.||||

EMBOSS_001 88 AGGCAGCCTCCGGTGGCGCGATAGCGCCAACGTTCTCAACAGGCGCCC-- 135

**(IX)**

EMBOSS_001 951 TACCTCGCGAATGCATCTAGATTCTCGACGTCAGCCTGGACTATATGGAA 1000

|||||||||||||||||||||||||||

EMBOSS_001 1 -----------------------CTCGACGTCAGCCTGGACTATATGGAA 27

EMBOSS_001 1001 TCGCTCGAGGAAAAAGACAAATCTGCCCTCAGAGCTTGAGAACATCTTCG 1050

||||||||||||||||||||||||||||||||||||||||||||||||||

EMBOSS_001 28 TCGCTCGAGGAAAAAGACAAATCTGCCCTCAGAGCTTGAGAACATCTTCG 77

EMBOSS_001 1051 GATGCAGAGGAGGCAGCCTCCGGTGGCGCGATAGCGCCAACGTTCTCAAC 1100

||||||||||||||||||||||||||||||||||||||||||||||||||

EMBOSS_001 78 GATGCAGAGGAGGCAGCCTCCGGTGGCGCGATAGCGCCAACGTTCTCAAC 127

EMBOSS_001 1101 AGGCGCCCAATCGGATCCCGGGCCCGTCGACTGCRGAGGCCTGCATGCAA 1150

||||||||

EMBOSS_001 128 AGGCGCCC------------------------------------------ 135

**(X)**

EMBOSS_001 951 CTAGATTCTCGACGTCAGCCTGGACTAATACGAATCGCTCGAGGAAAAAG 1000

|||||||||||||||||||||||||||||||||||||||||||

EMBOSS_001 1 -------CTCGACGTCAGCCTGGACTAATACGAATCGCTCGAGGAAAAAG 43

EMBOSS_001 1001 ACAAATCTGCCCTCAGAGCTTGAGAACATCTTCGGATGCAGAGGAGGCAG 1050

||||||||||||||||||||||||||||||||||||||||||||||||||

EMBOSS_001 44 ACAAATCTGCCCTCAGAGCTTGAGAACATCTTCGGATGCAGAGGAGGCAG 93

EMBOSS_001 1051 CCTCCGGTGGCGCGATAGCGCCAACGTTCTCAACAGGCGCCCAATCGGAT 1100

||||||||||||||||||||||||||||||||||||||||||

EMBOSS_001 94 CCTCCGGTGGCGCGATAGCGCCAACGTTCTCAACAGGCGCCC-------- 135

**(XI)**

EMBOSS_001 51 CGGGCCCGGGATCCGATTCYCSACKTCAGCCTGGACCATGGAGACTCACT 100

|.|.||.|||||||||||||||||||||||||

EMBOSS_001 1 ------------------CTCGACGTCAGCCTGGACCATGGAGACTCACT 32

EMBOSS_001 101 ATAGGAAAAAGACAAATCTGCCCTCAGAGCTTGAGAACATCTTCGGATGC 150

||||||||||||||||||||||||||||||||||||||||||||||||||

EMBOSS_001 33 ATAGGAAAAAGACAAATCTGCCCTCAGAGCTTGAGAACATCTTCGGATGC 82

EMBOSS_001 151 AGAGGAGGCAGCCTCCGGTGGCGCGATAGCGCCAACGTTCTCAACAGGCG 200

||||||||||||||||||||||||||||||||||||||||||||||||||

EMBOSS_001 83 AGAGGAGGCAGCCTCCGGTGGCGCGATAGCGCCAACGTTCTCAACAGGCG 132

EMBOSS_001 201 CCCAATCTAGATGCATTCGCGAGGTACCGAGCTCGAATTCACTGGCCGTC 250

|||

EMBOSS_001 133 CCC----------------------------------------------- 135

**(XII)**

EMBOSS_001 951 TTCGAGCTCGGTACCTCGCGAATGCATCTAGATTCTCGACGTCAGCCTGG 1000

||||||||||||||||

EMBOSS_001 1 ----------------------------------CTCGACGTCAGCCTGG 16

EMBOSS_001 1001 ACCATGGACCTCGCCTCAAGGAAAAAGACAAATCTGCCCTCAGAGCTTGA 1050

||||||||||||||||||||||||||||||||||||||||||||||||||

EMBOSS_001 17 ACCATGGACCTCGCCTCAAGGAAAAAGACAAATCTGCCCTCAGAGCTTGA 66

EMBOSS_001 1051 GAACATCTTCGGATGCAGAGGAGGCAGCCTCCGGTGGCGCGATAGCGCCA 1100

||||||||||||||||||||||||||||||||||||||||||||||||||

EMBOSS_001 67 GAACATCTTCGGATGCAGAGGAGGCAGCCTCCGGTGGCGCGATAGCGCCA 116

EMBOSS_001 1101 ACGTTCTCAACAGGSGCCCAATCGGATCCCGGGCCCGTCGACTGCAGAGG 1150

||||||||||||||.||||

EMBOSS_001 117 ACGTTCTCAACAGGCGCCC------------------------------- 135

## Section SD: Confirmation of self-ligation activity of R18-T3 RNAs by sequence analysis

The amplicons (110 bp in size) indicating self-ligation activity of R18-T3 RNA with each of the substrates 1, 2, 3, 4, 5, 6, 7, 6a, 6b, 7a, 7b, 8a, 8b were sequenced and aligned (top) with the expected sequence (below). Shown are sequence alignments of ligated product of R18-T3 with (I) oligonucleotide substrate 1 (II) oligonucleotide substrate 2 (III) oligonucleotide substrate 3 (IV) oligonucleotide substrate 4 (V) oligonucleotide substrate 5 (VI) oligonucleotide substrate 6 (VII) oligonucleotide substrate 7 (VIII) oligonucleotide substrate 6a (IX) oligonucleotide substrate 6b (X) oligonucleotide substrate 7a (XI) oligonucleotide substrate 7b (XII) oligonucleotide substrate 8a (XIII) oligonucleotide substrate 8b

**(I)**

EMBOSS_001 51 GACGGGCCCGGGATCCGATTCYCGACKTCAGCCTGGACTAATACGACTCA 100

|.||||.|||||||||||||||||||||||

EMBOSS_001 1 --------------------CTCGACGTCAGCCTGGACTAATACGACTCA 30

EMBOSS_001 101 CTATAGGAAAAAGACAAATCTGCCCTCAGAGCTTGAGAACATCTTCGGAT 150

||||||||||||||||||||||||||||||||||||||||||||||||||

EMBOSS_001 31 CTATAGGAAAAAGACAAATCTGCCCTCAGAGCTTGAGAACATCTTCGGAT 80

EMBOSS_001 151 GCAGAGGAGGCAGCCTCCGGTGGCGCGATAATCTAGATGCATTCGCGAGG 200

||||||||||||||||||||||||||||||

EMBOSS_001 81 GCAGAGGAGGCAGCCTCCGGTGGCGCGATA-------------------- 110

**(II)**

EMBOSS_001 951 CGAGCTCGGTACCTCGCGAATGCATCTAGATTGTCAACTTCCGCATGAAC 1000

||||||||||||||||||

EMBOSS_001 1 --------------------------------GTCAACTTCCGCATGAAC 18

EMBOSS_001 1001 GAATACTACGCACTAAAGGAAAAAGACAAATCTGCCCTCAGAGCTTGAGA 1050

||||||||||||||||||||||||||||||||||||||||||||||||||

EMBOSS_001 19 GAATACTACGCACTAAAGGAAAAAGACAAATCTGCCCTCAGAGCTTGAGA 68

EMBOSS_001 1051 ACATCTTCGGATGCAGAGGAGGCAGCCTCCGGTGGCGSGATAAATCGGAT 1100

|||||||||||||||||||||||||||||||||||||.||||

EMBOSS_001 69 ACATCTTCGGATGCAGAGGAGGCAGCCTCCGGTGGCGCGATA-------- 110

**(III)**

EMBOSS_001 51 ACGGGCCCGGGATCCGATTCACGACGACAACCTGGTCTAATACGCCTCAC 100

|||||||||||||||||||||||||||||||

EMBOSS_001 1 -------------------CACGACGACAACCTGGTCTAATACGCCTCAC 31

EMBOSS_001 101 GATAGGAAAAAGACAAATCTGCCCTCAGAGCTTGAGAACATCTTCGGATG 150

||||||||||||||||||||||||||||||||||||||||||||||||||

EMBOSS_001 32 GATAGGAAAAAGACAAATCTGCCCTCAGAGCTTGAGAACATCTTCGGATG 81

EMBOSS_001 151 CAGAGGAGGCAGCCTCCGGTGGCGCGATAAATCTAGATGCATTCGCGAGG 200

|||||||||||||||||||||||||||||

EMBOSS_001 82 CAGAGGAGGCAGCCTCCGGTGGCGCGATA--------------------- 110

**(IV)**

EMBOSS_001 51 GACGGGCCCGGGATCCGATTCTGGATGTAAGTCTTGAATATATGGAATCG 100

||||||||||||||||||||||||||||||

EMBOSS_001 1 --------------------CTGGATGTAAGTCTTGAATATATGGAATCG 30

EMBOSS_001 101 CTCGAGGAAAAAGACAAATCTGCCCTCAGAGCTTGAGAACATCTTCGGAT 150

||||||||||||||||||||||||||||||||||||||||||||||||||

EMBOSS_001 31 CTCGAGGAAAAAGACAAATCTGCCCTCAGAGCTTGAGAACATCTTCGGAT 80

EMBOSS_001 151 GCAGAGGAGGCAGCCTCCGGTGGCGCGATAAATCTAGATGCATTCGCGAG 200

||||||||||||||||||||||||||||||

EMBOSS_001 81 GCAGAGGAGGCAGCCTCCGGTGGCGCGATA-------------------- 110

**(V)**

EMBOSS_001 51 ACGGGCCCGGGATCCGATTTAATACTCATAACGACTACATGGACCTCGCC 100

|||||||||||||||||||||||||||||||

EMBOSS_001 1 -------------------TAATACTCATAACGACTACATGGACCTCGCC 31

EMBOSS_001 101 TCAAGGAAAAAGACAAATCTGCCCTCAGAGCTTGAGAACATCTTCGGATG 150

||||||||||||||||||||||||||||||||||||||||||||||||||

EMBOSS_001 32 TCAAGGAAAAAGACAAATCTGCCCTCAGAGCTTGAGAACATCTTCGGATG 81

EMBOSS_001 151 CAGAGGAGGCAGCCTCCGGTGGCGCGATAAATCTAGATGCATTCGCGAGG 200

|||||||||||||||||||||||||||||

EMBOSS_001 82 CAGAGGAGGCAGCCTCCGGTGGCGCGATA--------------------- 110

**(VI)**

EMBOSS_001 51 GACGGGCCCGGGATCCGATTCYCGACGTCAGCCTGGACTAATACTAAAAA 100

|.||||||||||||||||||||||||||||

EMBOSS_001 1 --------------------CTCGACGTCAGCCTGGACTAATACTAAAAA 30

EMBOSS_001 101 CTATAGGAAAAAGACAAATCTGCCCTCAGAGCTTGAGAACATCTTCGGAT 150

||||||||||||||||||||||||||||||||||||||||||||||||||

EMBOSS_001 31 CTATAGGAAAAAGACAAATCTGCCCTCAGAGCTTGAGAACATCTTCGGAT 80

EMBOSS_001 151 GCAGAGGAGGCAGCCTCCGGTGGCGCGATAATCTAGATGCATTCGCGAGG 200

||||||||||||||||||||||||||||||

EMBOSS_001 81 GCAGAGGAGGCAGCCTCCGGTGGCGCGATA-------------------- 110

**(VII)**

EMBOSS_001 51 GACGGGCCCGGGATCCGATTCTCGACGTCAGCCTGGACTAATACTATTTA 100

||||||||||||||||||||||||||||||

EMBOSS_001 1 --------------------CTCGACGTCAGCCTGGACTAATACTATTTA 30

EMBOSS_001 101 CTATAGGAAAAAGACAAATCTGCCCTCAGAGCTTGAGAACATCTTCGGAT 150

||||||||||||||||||||||||||||||||||||||||||||||||||

EMBOSS_001 31 CTATAGGAAAAAGACAAATCTGCCCTCAGAGCTTGAGAACATCTTCGGAT 80

EMBOSS_001 151 GCAGAGGAGGCAGCCTCCGGTGGCGCGATAATCTAGATGCATTCGCGAGG 200

||||||||||||||||||||||||||||||

EMBOSS_001 81 GCAGAGGAGGCAGCCTCCGGTGGCGCGATA-------------------- 110

**(VIII)**

EMBOSS_001 51 CGGGCCCGGGATCCGATTCTCGACGTCAGCCTGGACTAATACTAGGGACT 100

||||||||||||||||||||||||||||||||

EMBOSS_001 1 ------------------CTCGACGTCAGCCTGGACTAATACTAGGGACT 32

EMBOSS_001 101 ATAGGAAAAAGACAAATCTGCCCTCAGAGCTTGAGAACATCTTCGGATGC 150

||||||||||||||||||||||||||||||||||||||||||||||||||

EMBOSS_001 33 ATAGGAAAAAGACAAATCTGCCCTCAGAGCTTGAGAACATCTTCGGATGC 82

EMBOSS_001 151 AGAGGAGGCAGCCTCCGGTGGCGCGATAATCTAGATGCATTCGCGAGGTA 200

||||||||||||||||||||||||||||

EMBOSS_001 83 AGAGGAGGCAGCCTCCGGTGGCGCGATA---------------------- 110

**(IX)**

EMBOSS_001 951 GGTACCTCGCGAATGCATCTAGATTCTCGACGTCAGCCTGGACTATATGG 1000

|||||||||||||||||||||||||

EMBOSS_001 1 -------------------------CTCGACGTCAGCCTGGACTATATGG 25

EMBOSS_001 1001 ACTCACTATAGGAAAAAGACAAATCTGCCCTCAGAGCTTGAGAACATCTT 1050

||||||||||||||||||||||||||||||||||||||||||||||||||

EMBOSS_001 26 ACTCACTATAGGAAAAAGACAAATCTGCCCTCAGAGCTTGAGAACATCTT 75

EMBOSS_001 1051 CGGATGCAGAGGAGGCAGCCTCCGGTGGCGCGATAAATCGGATCCCGGGC 1100

|||||||||||||||||||||||||||||||||||

EMBOSS_001 76 CGGATGCAGAGGAGGCAGCCTCCGGTGGCGCGATA--------------- 110

**(X)**

EMBOSS_001 951 ATTCGAGCTCGGTACCTCGCGAATGCATCTAGATTCTCGACGTCAGCCTG 1000

|||||||||||||||

EMBOSS_001 1 -----------------------------------CTCGACGTCAGCCTG 15

EMBOSS_001 1001 GACTATATGGAATCGCTCGAGGAAAAAGACAAATCTGCCCTCAGAGCTTG 1050

||||||||||||||||||||||||||||||||||||||||||||||||||

EMBOSS_001 16 GACTATATGGAATCGCTCGAGGAAAAAGACAAATCTGCCCTCAGAGCTTG 65

EMBOSS_001 1051 AGAACATCTTCGGATGCAGAGGAGGCAGCCTCCGGTGGCGCGATAAATCG 1100

|||||||||||||||||||||||||||||||||||||||||||||

EMBOSS_001 66 AGAACATCTTCGGATGCAGAGGAGGCAGCCTCCGGTGGCGCGATA----- 110

**(XI)**

EMBOSS_001 51 ACGGGCCCGGGATCCGATTCYCGACGTCAGCCTGGACTAATACGAATCGC 100

|.|||||||||||||||||||||||||||||

EMBOSS_001 1 -------------------CTCGACGTCAGCCTGGACTAATACGAATCGC 31

EMBOSS_001 101 TCGAGGAAAAAGACAAATCTGCCCTCAGAGCTTGAGAACATCTTCGGATG 150

||||||||||||||||||||||||||||||||||||||||||||||||||

EMBOSS_001 32 TCGAGGAAAAAGACAAATCTGCCCTCAGAGCTTGAGAACATCTTCGGATG 81

EMBOSS_001 151 CAGAGGAGGCAGCCTCCGGTGGCGCGATAATCTAGATGCATTCGCGAGGT 200

|||||||||||||||||||||||||||||

EMBOSS_001 82 CAGAGGAGGCAGCCTCCGGTGGCGCGATA--------------------- 110

**(XII)**

EMBOSS_001 901 CTCGACGTCAGCCTGGACCATGGAGACTCACTATAGGAAAAAGACAAATC 950

||||||||||||||||||||||||||||||||||||||||||||||||||

EMBOSS_001 1 CTCGACGTCAGCCTGGACCATGGAGACTCACTATAGGAAAAAGACAAATC 50

EMBOSS_001 951 TGCCCTCAGAGCTTGAGAACATCTTCGGATGCAGAGGAGGCAGCCTCCGK 1000

|||||||||||||||||||||||||||||||||||||||||||||||||.

EMBOSS_001 51 TGCCCTCAGAGCTTGAGAACATCTTCGGATGCAGAGGAGGCAGCCTCCGG 100

EMBOSS_001 1001 TKKCGCGATAAATCGGATCCCGGGCCCGTCGACTGSAGAGGCCTGCATKC 1050

|..|||||||

EMBOSS_001 101 TGGCGCGATA---------------------------------------- 110

**(XIII)**

EMBOSS_001 51 GGGCCCGGGATCCGATTCTCGACGTCAGCCTGGACTAATACCCTCGCCTC 100

|||||||||||||||||||||||||||||||||

EMBOSS_001 1 -----------------CTCGACGTCAGCCTGGACTAATACCCTCGCCTC 33

EMBOSS_001 101 AAGGAAAAAGACAAATCTGCCCTCAGAGCTTGAGAACATCTTCGGATGCA 150

||||||||||||||||||||||||||||||||||||||||||||||||||

EMBOSS_001 34 AAGGAAAAAGACAAATCTGCCCTCAGAGCTTGAGAACATCTTCGGATGCA 83

EMBOSS_001 151 GAGGAGGCAGCCTCCGGTGGCGCGATAAATCTAGATGCATTCGCGAGGTA 200

|||||||||||||||||||||||||||

EMBOSS_001 84 GAGGAGGCAGCCTCCGGTGGCGCGATA----------------------- 110

**Section SE: Confirmation of self-ligation activity of R18-T4 RNAs by sequence analysis**

The amplicons (75 bp in size) indicating self-ligation activity of R18-T4 RNA with each of the substrates 1, 2, 3, 4, 5, 6, 7, 6a, 6b, 7a, 7b, 8a, 8b were sequenced (top) and aligned with the expected sequence (below). Shown are sequence alignments of ligated product of R18-T4 with (I) oligonucleotide substrate 1 (II) oligonucleotide substrate 2 (III) oligonucleotide substrate 3 (IV) oligonucleotide substrate 4 (V) oligonucleotide substrate 5 (VI) oligonucleotide substrate 6 (VII) oligonucleotide substrate 7 (VIII) oligonucleotide substrate 6a (IX) oligonucleotide substrate 6b (X) oligonucleotide substrate 7a (XI) oligonucleotide substrate 7b (XII) oligonucleotide substrate 8a (XIII) oligonucleotide substrate 8b

**(I)**

EMBOSS_001 51 GACGGGCCCGGGATCCGATTCYCGACGTCAGCCTGGACTAATACGACTCA 100

|.||||||||||||||||||||||||||||

EMBOSS_001 1 --------------------CTCGACGTCAGCCTGGACTAATACGACTCA 30

EMBOSS_001 101 CTATAGGAAAAAGACAAATCTGCCCTCAGAGCTTGAGAACATCTTAATCT 150

|||||||||||||||||||||||||||||||||||||||||||||

EMBOSS_001 31 CTATAGGAAAAAGACAAATCTGCCCTCAGAGCTTGAGAACATCTT----- 75

**(II)**

EMBOSS_001 1001 TCGCGAATGCATCTAGATTGTCAACTTCCGCATGAACGAATACTACGCAC 1050

|||||||||||||||||||||||||||||||

EMBOSS_001 1 -------------------GTCAACTTCCGCATGAACGAATACTACGCAC 31

EMBOSS_001 1051 TAAAGGAAAAAGACAAATCTGCCCTCAGAGCTTGAGAACATCTTAATCGG 1100

||||||||||||||||||||||||||||||||||||||||||||

EMBOSS_001 32 TAAAGGAAAAAGACAAATCTGCCCTCAGAGCTTGAGAACATCTT------ 75

**(III)**

EMBOSS_001 51 ACGGGCCCGGGATCCGATTCACGACGACAACCTGGTCTAATACGCCTCAC 100

|||||||||||||||||||||||||||||||

EMBOSS_001 1 -------------------CACGACGACAACCTGGTCTAATACGCCTCAC 31

EMBOSS_001 101 GATAGGAAAAAGACAAATCTGCCCTCAGAGCTTGAGAACATCTTAATCTA 150

||||||||||||||||||||||||||||||||||||||||||||

EMBOSS_001 32 GATAGGAAAAAGACAAATCTGCCCTCAGAGCTTGAGAACATCTT------

**(IV)**

EMBOSS_001 51 ACGGGCCCGGGATCCGATTCTGGATGTAAGTCTTGAATATATGGAATCGC 100

|||||||||||||||||||||||||||||||

EMBOSS_001 1 -------------------CTGGATGTAAGTCTTGAATATATGGAATCGC 31

EMBOSS_001 101 TCGAGGAAAAAGACAAATCTGCCCTCAGAGCTTGAGAACATCTTAATCTA 150

||||||||||||||||||||||||||||||||||||||||||||

EMBOSS_001 32 TCGAGGAAAAAGACAAATCTGCCCTCAGAGCTTGAGAACATCTT------ 75

**(V)**

EMBOSS_001 51 ACGGGCCCGGGATCCGATTTAATACTCATAACGACTACATGGACCTCGCC 100

|||||||||||||||||||||||||||||||

EMBOSS_001 1 -------------------TAATACTCATAACGACTACATGGACCTCGCC 31

EMBOSS_001 101 TCAAGGAAAAAGACAAATCTGCCCTCAGAGCTTGAGAACATCTTAATCTA 150

||||||||||||||||||||||||||||||||||||||||||||

EMBOSS_001 32 TCAAGGAAAAAGACAAATCTGCCCTCAGAGCTTGAGAACATCTT------ 75

**(VI)**

EMBOSS_001 1001 GCATCTAGATTCTCGACGTCAGCCTGGACTAATACTAAAAACTATAGGAA 1050

|||||||||||||||||||||||||||||||||||||||

EMBOSS_001 1 -----------CTCGACGTCAGCCTGGACTAATACTAAAAACTATAGGAA 39

EMBOSS_001 1051 AAAGACAAATCTGCCCTCAGAGCTTGAGAACATCTTAATCGGATCCCGGG 1100

||||||||||||||||||||||||||||||||||||

EMBOSS_001 40 AAAGACAAATCTGCCCTCAGAGCTTGAGAACATCTT-------------- 75

**(VII)**

EMBOSS_001 1001 TGCATCTAGATTCGACGTCAGCCTGGACTAATACTATTTACTATAGGAAA 1050

.|||||||||||||||||||||||||||||||||||||||

EMBOSS_001 1 ----------CTCGACGTCAGCCTGGACTAATACTATTTACTATAGGAAA 40

EMBOSS_001 1051 AAGACAAATCTGCCCTCAGAGCTTGAGAACAKCTTATCGGATCCCGGGCC 1100

|||||||||||||||||||||||||||||||.|||

EMBOSS_001 41 AAGACAAATCTGCCCTCAGAGCTTGAGAACATCTT--------------- 75

**(VIII)**

EMBOSS_001 51 CGGGCCCGGGATCCGATTCTCGACGTCAGCCTGGACTAATACTAGGGACT 100

||||||||||||||||||||||||||||||||

EMBOSS_001 1 ------------------CTCGACGTCAGCCTGGACTAATACTAGGGACT 32

EMBOSS_001 101 ATAGGAAAAAGACAAATCTGCCCTCAGAGCTTGAGAACATCTTAATCTAG 150

|||||||||||||||||||||||||||||||||||||||||||

EMBOSS_001 33 ATAGGAAAAAGACAAATCTGCCCTCAGAGCTTGAGAACATCTT------- 75

**(IX)**

EMBOSS_001 51 KTCGACGGGCCCGGGATCCGATTCTCGACGTCAGCCTGGACTATATGGAC 100

|||||||||||||||||||||||||||

EMBOSS_001 1 -----------------------CTCGACGTCAGCCTGGACTATATGGAC 27

EMBOSS_001 101 TCACTATAGGAAAAAGACAAATCTGCCCTCAGAGCTTGAGAACATCTTAA 150

||||||||||||||||||||||||||||||||||||||||||||||||

EMBOSS_001 28 TCACTATAGGAAAAAGACAAATCTGCCCTCAGAGCTTGAGAACATCTT-- 75

**(X)**

EMBOSS_001 1001 ATTCGAGCTCGGTACCTCGCGAATGCATCTAGATTCTCGACGTCAGCCTG 1050

|||||||||||||||

EMBOSS_001 1 -----------------------------------CTCGACGTCAGCCTG 15

EMBOSS_001 1051 GACTATATGGAATCGCTCGAGGAAAAAGACAAATCTGCCCTCAGAGCTTG 1100

||||||||||||||||||||||||||||||||||||||||||||||||||

EMBOSS_001 16 GACTATATGGAATCGCTCGAGGAAAAAGACAAATCTGCCCTCAGAGCTTG 65

EMBOSS_001 1101 AGAACATCTTAATCGGATCCCGGGCCCGTCGACTGCRGAGGCCTGCATGC 1150

||||||||||

EMBOSS_001 66 AGAACATCTT---------------------------------------- 75

**(XI)**

EMBOSS_001 1001 TCGAGCTCGGTACCTCGCGAATGCATCTAGATTCTCGACGTCAGCCTGGA 1050

|||||||||||||||||

EMBOSS_001 1 ---------------------------------CTCGACGTCAGCCTGGA 17

EMBOSS_001 1051 CTAATACGAATCGCTCGAGGAAAAAGACAAATCTGCCCTCAGAGCTTGAG 1100

||||||||||||||||||||||||||||||||||||||||||||||||||

EMBOSS_001 18 CTAATACGAATCGCTCGAGGAAAAAGACAAATCTGCCCTCAGAGCTTGAG 67

EMBOSS_001 1101 AACATCTTAATCGGATCCCGGGCCCGTCGACTGCRGAGGCCTGCATGCAA 1150

||||||||

EMBOSS_001 68 AACATCTT------------------------------------------ 75

**(XII)**

EMBOSS_001 51 GACGGGCCCGGGATCCGATTCTCGACGTCAGCCTGGACCATGGAGACTCA 100

||||||||||||||||||||||||||||||

EMBOSS_001 1 --------------------CTCGACGTCAGCCTGGACCATGGAGACTCA 30

EMBOSS_001 101 CTATAGGAAAAAGACAAATCTGCCCTCAGAGCTTGAGAACATCTTAATCT 150

|||||||||||||||||||||||||||||||||||||||||||||

EMBOSS_001 31 CTATAGGAAAAAGACAAATCTGCCCTCAGAGCTTGAGAACATCTT----- 75

**(XIII)**

EMBOSS_001 51 CGACGGGCCCGGGATCCGATTCTCGAYGTCAGCCTGGACTAATACCCTCG 100

|||||.|||||||||||||||||||||||

EMBOSS_001 1 ---------------------CTCGACGTCAGCCTGGACTAATACCCTCG 29

EMBOSS_001 101 CCTCAAGGAAAAAGACAAATCTGCCCTCAGAGCTTGAGAACATCTTAATC 150

||||||||||||||||||||||||||||||||||||||||||||||

EMBOSS_001 30 CCTCAAGGAAAAAGACAAATCTGCCCTCAGAGCTTGAGAACATCTT---- 75

**Section SF: Standard Curves used for quantification of ribozymes ligation activity.**

SF.1: Preparation of samples for standard curves

Standard curves were generated with crossing point (CP) values obtained from known copies of PCR amplified DNA of each of the ribozymes R18, R18-T1, R18-T2, R18-T3 and R18-T4 ligated to substrate 1. This was done as follows: Sequenced plasmid construct cloned with ligation product of each ribozyme with substrate 1 was PCR amplified using the respective forward and reverse primers as given in Table S2. Briefly, PCR was carried out using *Pfu* DNA polymerase with 25 pmoles of each forward and reverse primers in a total volume of 50 µl with the following thermocycling conditions: Initial denaturation at 95 ºC for 5 mins, 30 cycles of [denaturation at 95 ºC for 1 min, annealing at 58 ºC for 30 sec, extension at 72 ºC for 30 sec], and final extension at 72 ºC for 10 mins. The amplified product was run on a 2.5% agarose gel (Lonza, USA) and was purified to homogeneity using the NucleoSpin Extract II kit (Macherey-Nagel, Germany). It was sequenced for confirmation. The concentration of the gel purified and sequenced PCR product was determined using the Qubit dsDNA HS Assay kit according to the manufacturer’s instructions (Life technologies, USA). Serial dilutions of the DNA were prepared in nuclease free water to obtain copy numbers corresponding to a final concentration as given in Table 2.3. The amounts of DNA needed for obtaining X number of DNA copies was calculated as follows: Amount of DNA (in gms) = Moles (X number of Copies / 6.023 x 10^23^) x Molecular weight of DNA (gms/mol)

| Sample Name | No. of copies per µl |
| --- | --- |
| E | 1x10^7^ |
| F | 1x10^6^ |
| G | 1x10^5^ |
| H | 1x10^4^ |
| I | 1x10^3^ |
| J | 1x10^2^ |
| K | 1x10^1^ |

Table 2.3: The copies of DNA prepared for generating the standard curves.

Samples E-K are the dilutions of DNA prepared with the corresponding number of copies per µl

SF.2: Generation of standard curves

Standard curves were generated using log base 10 of initial target copy number (X axis) versus corresponding crossing point (CP) values obtained (Y axis). Crossing point values were obtained from samples labelled E, F, G, H, I, J and K corresponding to dilutions of 10^7^, 10^6^, 10^5^, 10^4^, 10^3^, 10^2^, 10 copies of DNA per µl respectively (shown in the table below each curve). A sample with no DNA was also prepared. (I) Standard curve of DNA copies of R18 ligated to substrate 1 (II) Standard curve of DNA copies of R18-T1 ligated to substrate 1 (III) Standard curve of DNA copies of R18-T2 ligated to substrate 1 (IV) Standard curve of DNA copies of R18-T3 ligated to substrate 1 (V) Standard curve of DNA copies of R18-T4 ligated to substrate 1

**(I)**


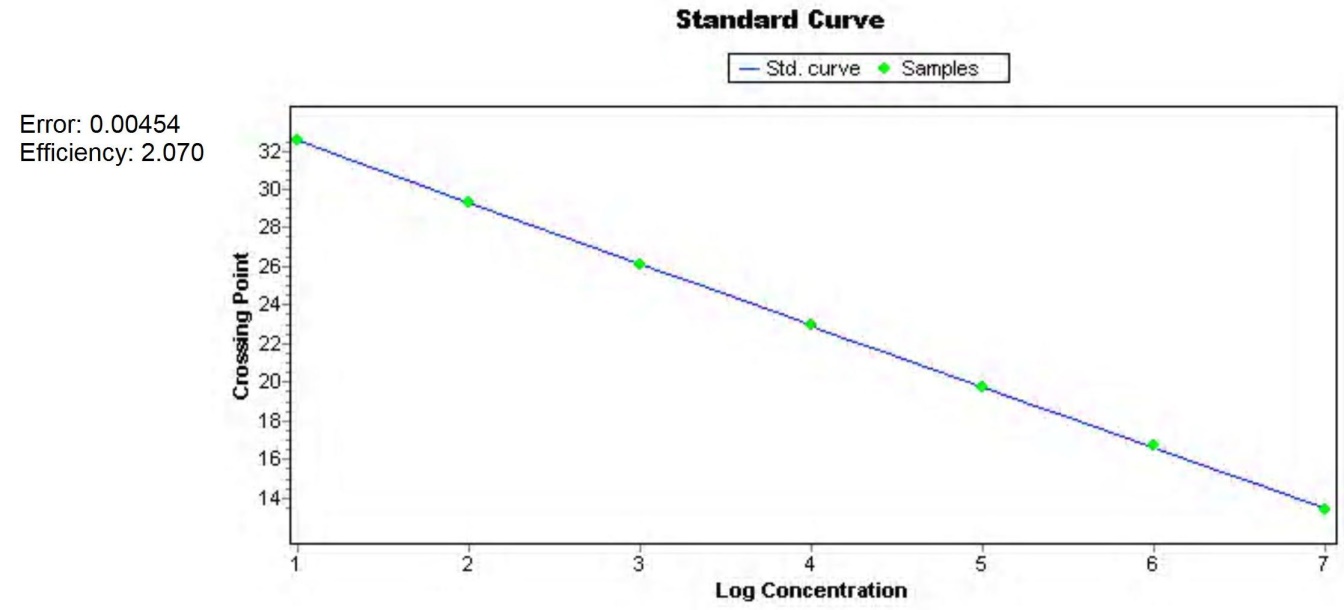


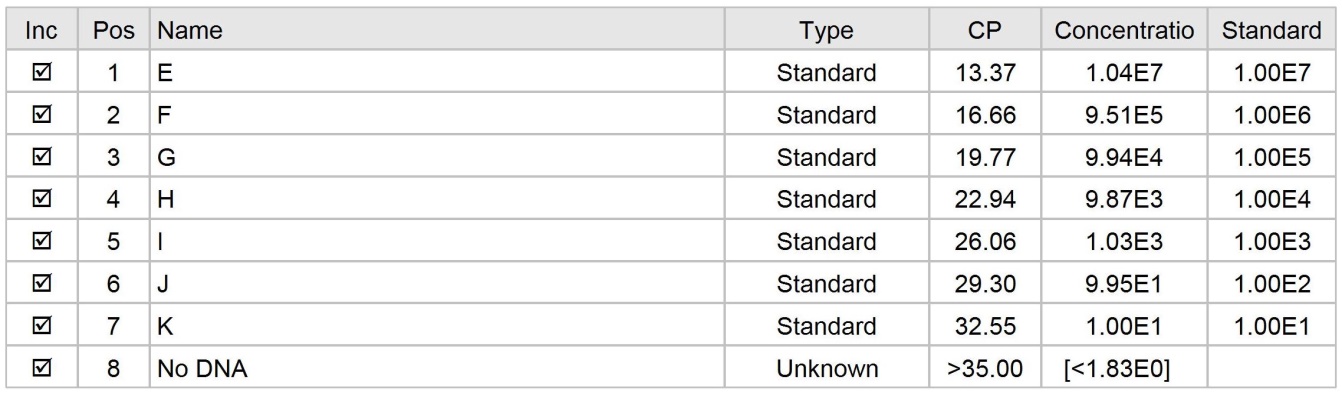


**(II)**


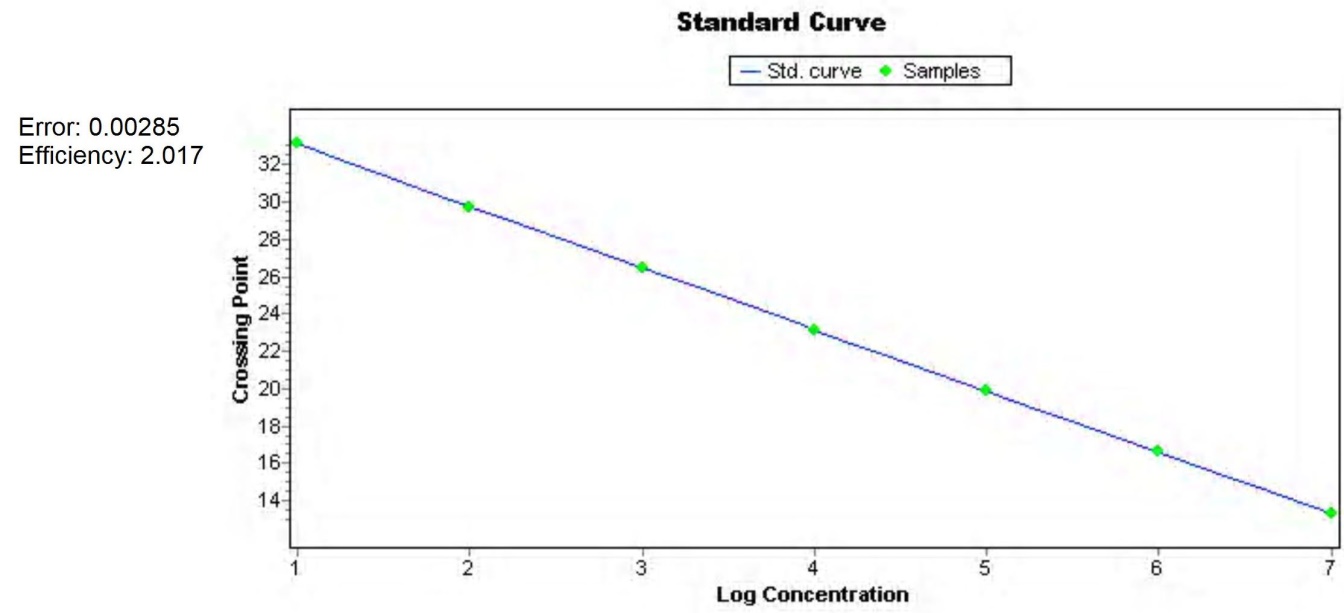


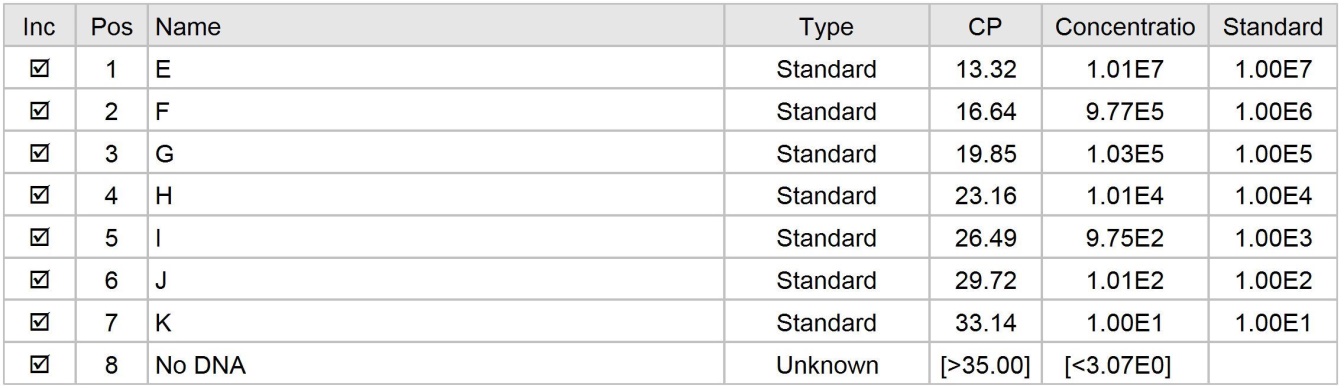


**(III)**


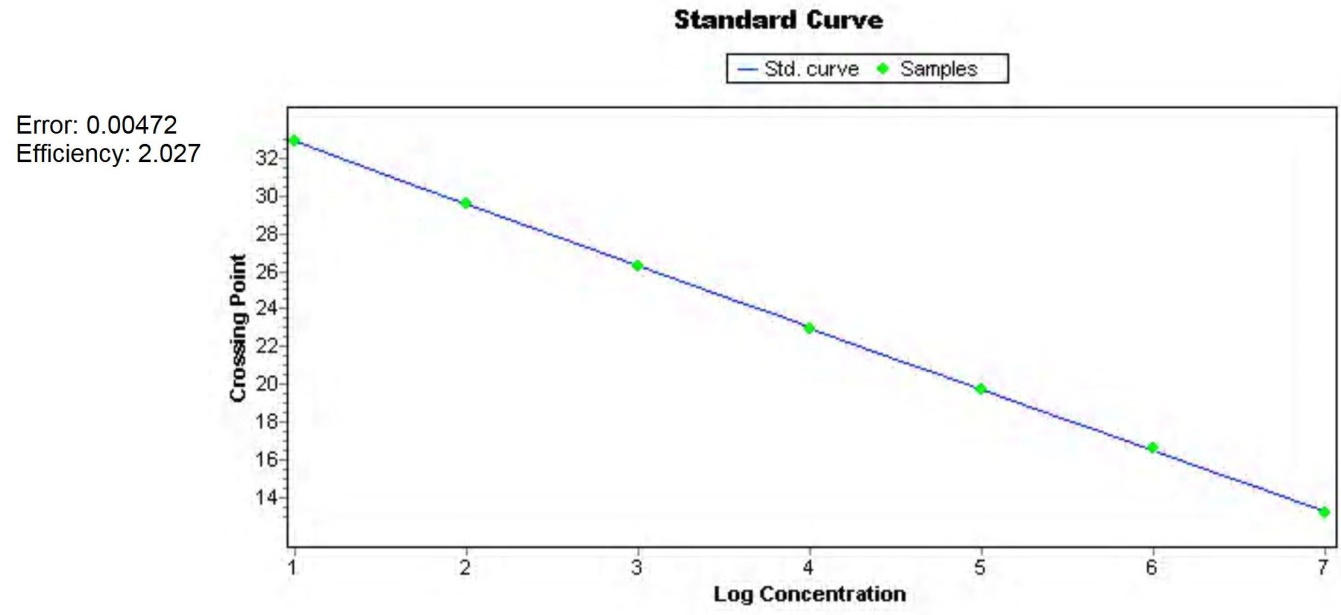


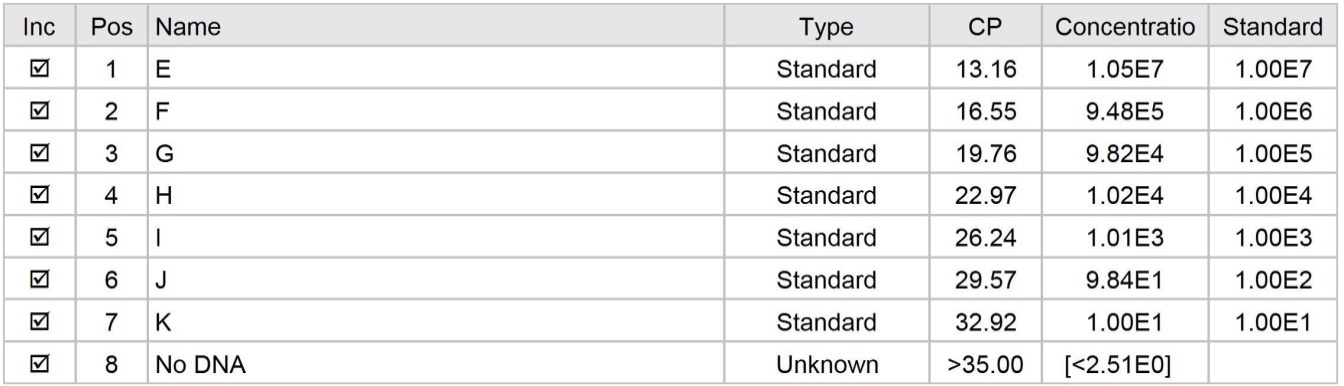


**(IV)**


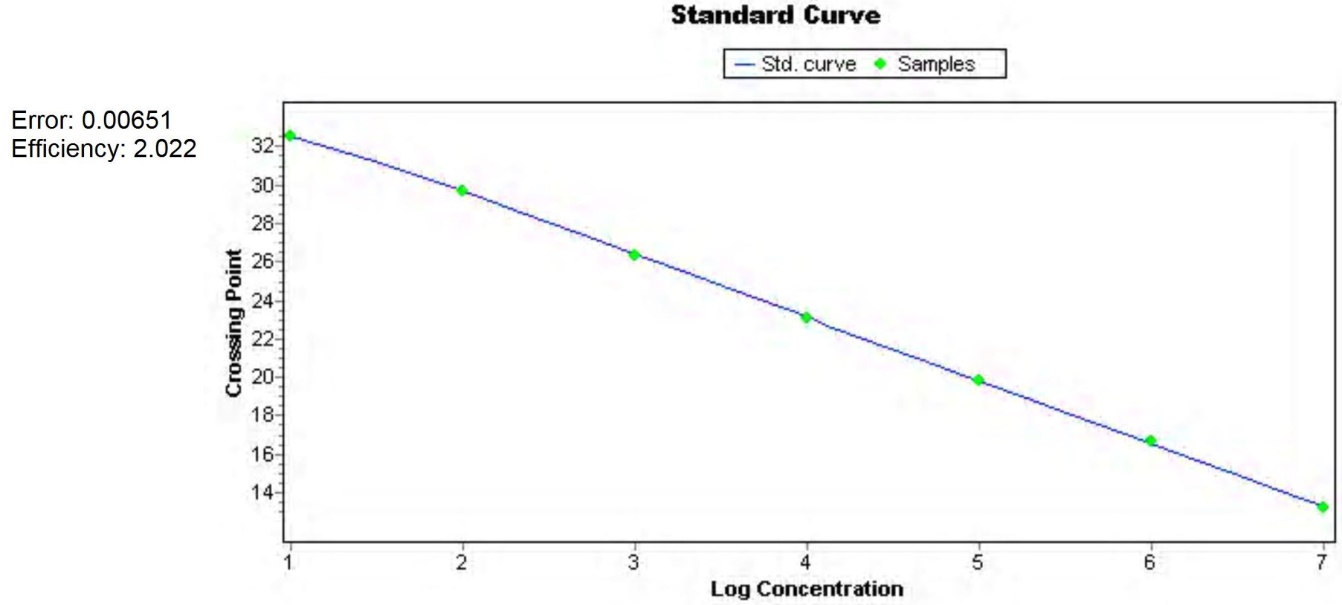


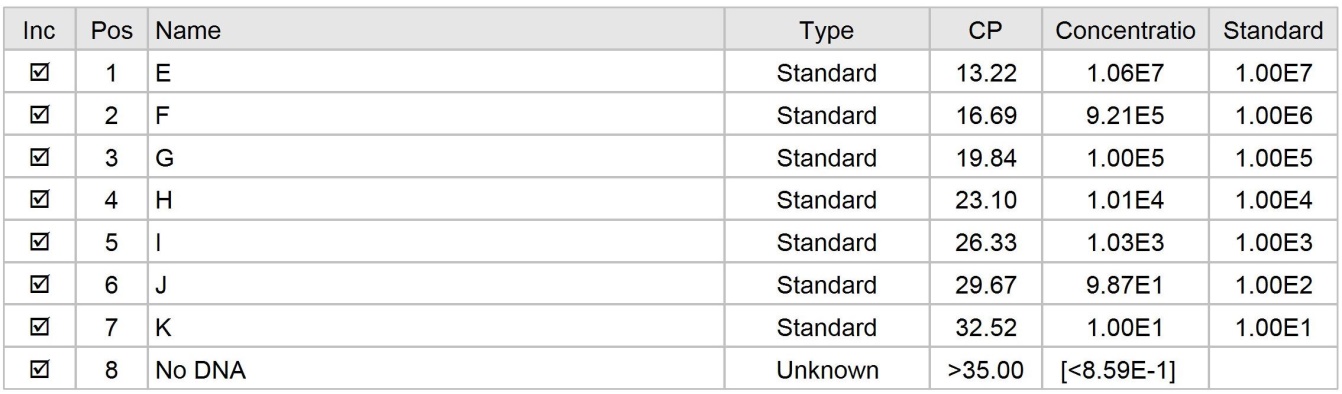


**(V)**


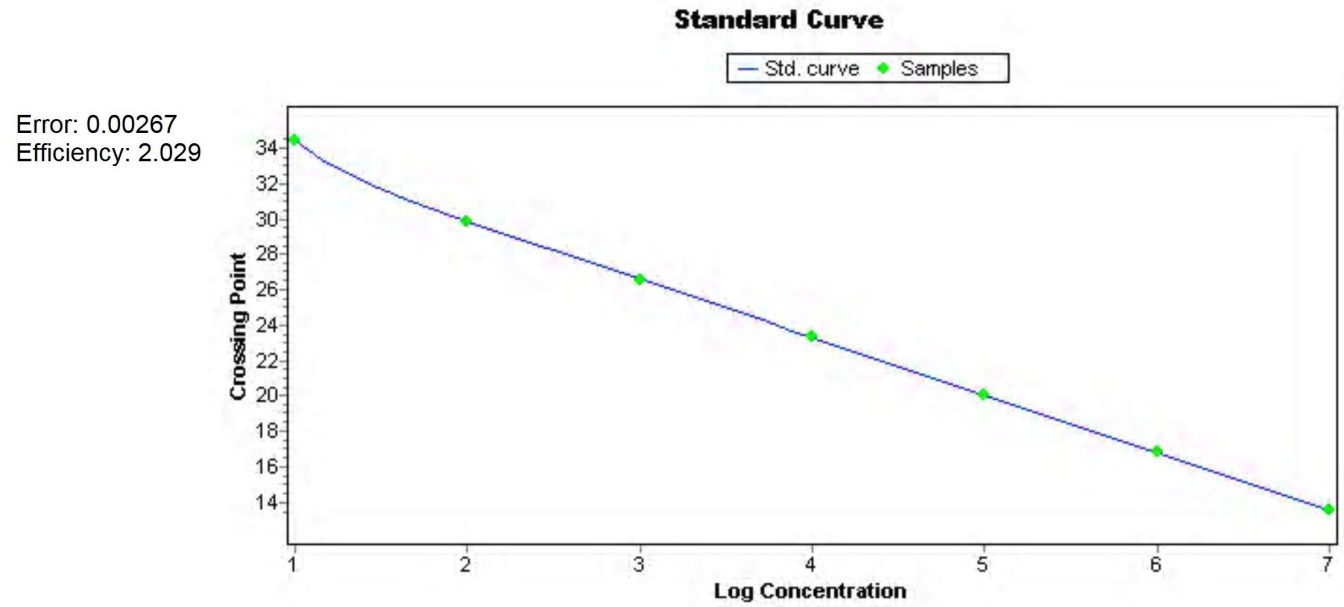


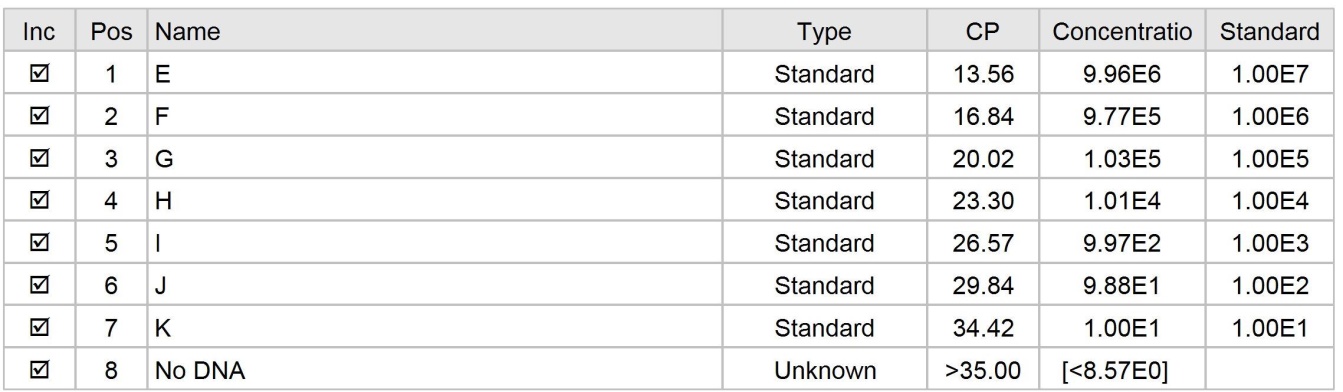

Supplement: Supplementary Figures providing detailed experimental protocols and methodologies [file rsos170376supp2.docx]
